# Iodine‐Catalysed Dissolution of Elemental Gold in Ethanol

**DOI:** 10.1002/anie.202117587

**Published:** 2022-02-15

**Authors:** Anže Zupanc, Eeva Heliövaara, Karina Moslova, Aleksi Eronen, Marianna Kemell, Črtomir Podlipnik, Marjan Jereb, Timo Repo

**Affiliations:** ^1^ Department of Chemistry Faculty of Science University of Helsinki A. I. Virtasen aukio 1 00014 Helsinki Finland; ^2^ Faculty of Chemistry and Chemical Technology University of Ljubljana Večna pot 113 1000 Ljubljana Slovenia

**Keywords:** Catalysis, Gold, Iodine, Recycling, Sustainable Chemistry

## Abstract

Gold is a scarce element in the Earth's crust but indispensable in modern electronic devices. New, sustainable methods of gold recycling are essential to meet the growing eco‐social demand of gold. Here, we describe a simple, inexpensive, and environmentally benign dissolution of gold under mild conditions. Gold dissolves quantitatively in ethanol using 2‐mercaptobenzimidazole as a ligand in the presence of a catalytic amount of iodine. Mechanistically, the dissolution of gold begins when I_2_ oxidizes Au^0^ and forms a [Au^I^I_2_]^−^ species, which undergoes subsequent ligand‐exchange reactions and forms a stable bis‐ligand Au^I^ complex. H_2_O_2_ oxidizes free iodide and regenerated I_2_ returns back to the catalytic cycle. Addition of a reductant to the reaction mixture precipitates gold quantitatively and partially regenerates the ligand. We anticipate our work will open a new pathway to more sustainable metal recycling with the utilization of just catalytic amounts of reagents and green solvents.

## Graphical abstract

Gold can be dissolved in a sustainable manner with common household chemicals: iodine solution in ethanol, resembling diluted iodine tincture, green oxidant hydrogen peroxide and 2‐mercaptobenzimidazole, a compound used in medicinal applications. Catalytic reactions and green solvents are the basis for sustainable development and recycling of precious metals like gold.

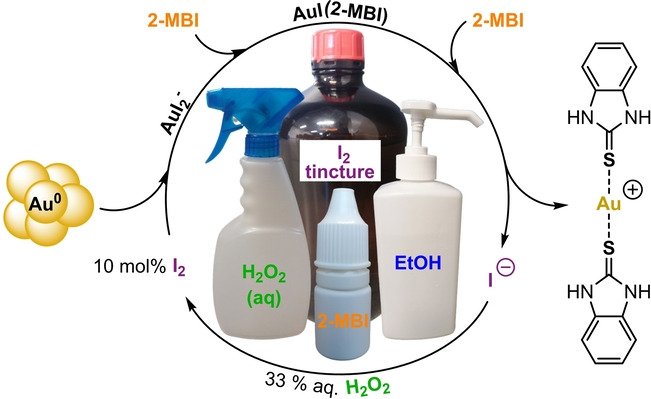

## Introduction

Noble metals have attractive chemical and physical properties, including good electrical and thermal conductivity, combined with resistance to oxidative stress. In particular, gold plays an invaluable role in modern microelectronics, including ubiquitous cell phones and computers.[^1^, ^2^, ^3^, ^4^, ^5^, ^6^, ^7^, ^8^] Demand for gold and other precious metals is expected to grow continuously in these and many other applications, including medicine,^[9]^ the chemical industry,[^10^, ^11^] and the space industry,^[12]^ among others. Due to the limited resources of noble metals in Earth's crust, recycling[^2^, ^3^, ^4^, ^5^, ^6^, ^7^, ^8^, ^13^, ^14^, ^15^] will have to accompany the traditional mineral extractions.[^16^, ^17^] Circular economy is imperative for their sustainable use.

**Figure 1:**
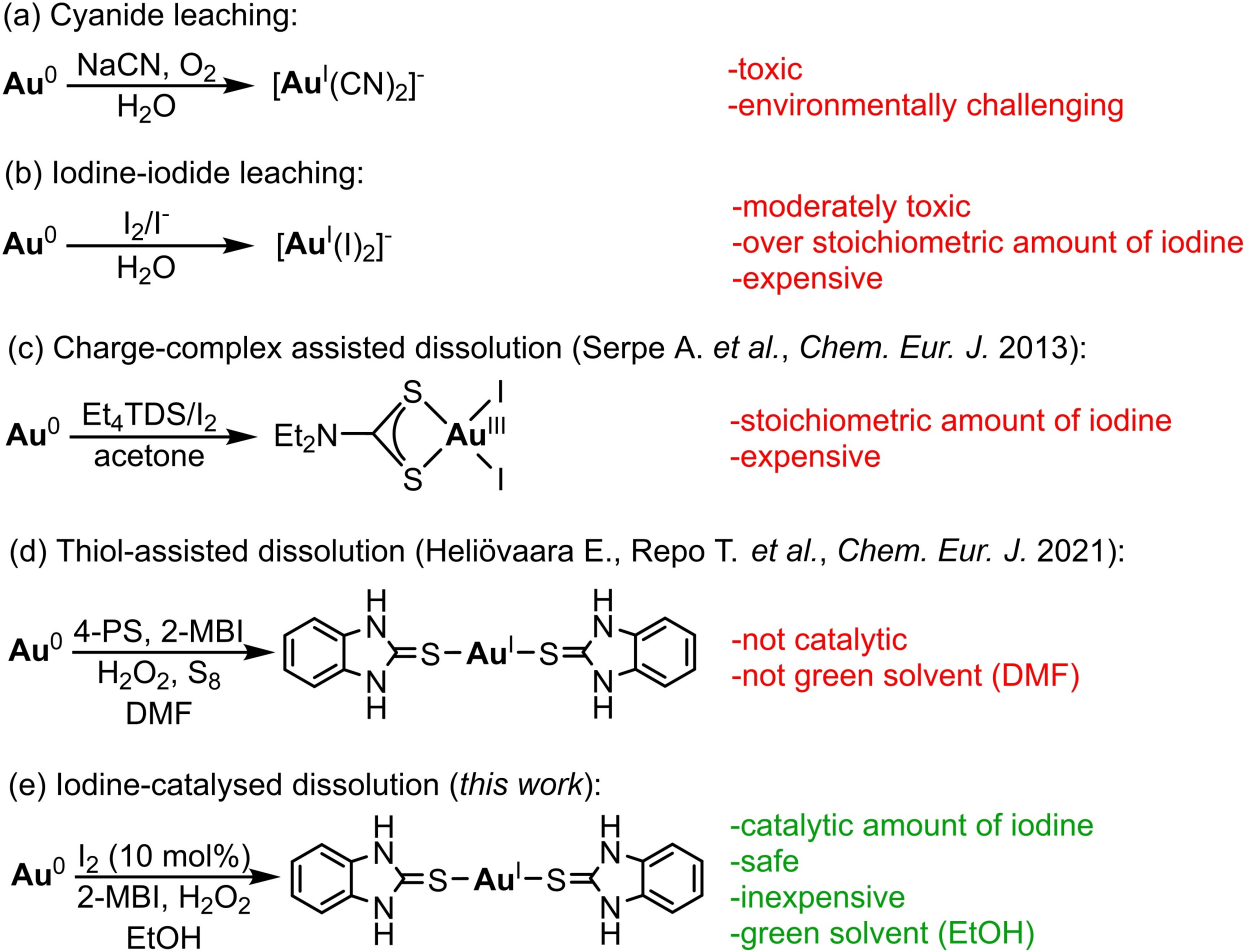
Different approaches to dissolving gold in water or in organic solvents. Gold can be dissolved with various methods that are accompanied with different problems related to sustainability of the process. Widely used cyanide leaching (a) is environmentally burdensome and toxic. More than stoichiometric amounts of moderately toxic iodine are needed for quantitative dissolution of gold in water (b, iodine–iodide leaching) or in organic solvents (c), which makes these methods less affordable. Expensive ligands in excess amounts (d) are often needed to dissolve gold in not necessarily sustainable solvents (DMF). Our new approach (e) combines catalytic amount of iodine and inexpensive compounds to dissolve gold in green solvent (EtOH), which makes this method safe and sustainable.

Main established Au recovery processes include environmentally challenging and toxic methods, such as cyanidation[^4^, ^6^, ^16^, ^17^, ^18^, ^19^] (Figure 1, a), and in less developed regions, even the amalgam process.^[20]^ Other dissolution procedures,[^4^, ^5^, ^6^, ^16^, ^17^, ^18^, ^19^] including aqueous iodine‐iodide leaching[^5^, ^6^, ^7^, ^8^, ^16^, ^17^, ^18^, ^19^, ^21^, ^22^, ^23^, ^24^, ^25^, ^26^, ^27^, ^28^, ^29^, ^30^, ^31^] (Figure 1, b), come with marked improvements in safety and environment issues. As previously reported, elemental halogens can oxidize Au^0^ to Au^I^ or Au^III^ cations and stabilize the formed Au cations in the solution as Lewis bases.[^32^, ^33^] Nevertheless, in addition to the moderate toxicity and costly production, over stoichiometric amounts of iodine are usually needed to afford a quantitative dissolution of Au, hence making this procedure less sustainable and less financially feasible.[^5^, ^16^, ^18^, ^21^, ^34^] Even with the assist of more environmentally benign oxidants, especially aqueous H_2_O_2_,[^5^, ^6^, ^19^, ^21^, ^23^, ^24^, ^25^, ^26^, ^27^] large amounts of iodine are consumed in the processes.

Dissolution of noble metals in organic solvents is a rather unexploited concept with inherent opportunity for improved selectivity and sustainability. For example, *organic aqua regia*[^35^, ^36^, ^37^] is highly selective, but it is still based on noxious SOCl_2_. Elemental halogens (I_2_, Br_2_) are also introduced to the concept as safer oxidants. Commonly, halogens are coupled in a charge‐complex[^34^, ^38^, ^39^, ^40^, ^41^, ^42^, ^43^, ^44^, ^45^, ^46^] (Figure 1, c) with various *S*‐donor ligands, in particular, dithiooxamides,[^38^, ^39^, ^40^, ^41^, ^42^] dithiocarbamates,[^42^, ^43^, ^44^] thiourea[^44^, ^45^] and thioimidophosphinic acid^[46]^ compounds. The latter then stabilize Au^III^ and Au^I^ centres in THF,[^38^, ^39^, ^40^, ^41^] MeCN,[^41^, ^47^, ^48^] DCM,[^45^, ^48^] chloroform,^[39]^ DMSO,^[49]^ acetone,[^41^, ^42^, ^43^] MEK,^[41]^ Et_2_O,[^44^, ^46^] ionic liquids,[^50^, ^51^] deep eutectic solvent,^[52]^ or even in water.^[34]^ Similar to aqueous iodine‐iodine leaching, over stoichiometric amounts of halogen are needed for quantitative dissolution of Au. However, the choice of a complex ligand is an additional barrier for these methods to reach large scale.

Previously, we reported a phenomenon that gold dissolves in ethanol in the presence of O_2_ and 4‐pyridinethione (4‐PS).^[53]^ Dissolution rates were modest, but as shown later, they are markedly improved by using H_2_O_2_ as oxidant and DMF as solvent. However, large amounts of expensive 4‐PS (200 equivalents compared to Au powder) are needed for quantitating the reaction.^[54]^ To further improve the approach, we have recently developed a novel strategy based on the ligand exchange on Au^I^ cation. By introducing inexpensive 2‐mercaptobenzimidazole (2‐MBI), a safe compound used in medicinal applications,[^55^, ^56^, ^57^, ^58^] the total amount of thiols is dramatically reduced (Figure 1, d).^[59]^ When dealing with complex metal mixtures, sequential dissolution seems like a realistic solution. By first removing the larger contents of metals with lower oxidation potential (Cu), gold can be selectively extracted from electronic waste material.^[54]^ However, there is still room for further improvements, particularly for those including catalytic reactions and green solvents.

In this respect, we designed a novel dissolution approach based on catalytic amount of iodine and 2‐MBI. This approach provides an environmentally benign way to dissolve Au quantitatively using EtOH as a green solvent and aqueous H_2_O_2_ as an oxidant. Under these reaction conditions, iodide is re‐oxidized to I_2_. This green strategy allows the use of catalytic amounts of I_2_ in gold dissolution for the first time and the concept is likely expandable for other metals.

## Results and Discussion

After a vast optimization of reaction parameters, we concluded that Au powder (2 mg for standard experiment) can be quantitatively dissolved in EtOH (10 mL) using 2‐MBI (20 equiv), 33 % aqueous H_2_O_2_ (20 equiv), and I_2_ (10 mol %) (see Supporting Information for details, Tables S1–S7). The initial concentration of I_2_ in ethanol is 0.003 %, and the solution resembles a diluted, commonly used antiseptic, iodine tincture. The reaction took place at 60 °C, and Au was dissolved quantitatively after 13 h (Figure 3). Key evidence of cooperation between ligand, oxidant, and catalyst is summarized in Table 1. Notably, 2‐MBI, together with H_2_O_2_, dissolves 48 % of Au (Table 1, entry 2) which is in accordance with the previously outlined reaction mechanism[^53^, ^54^, ^59^] and the proportion does not rise after a prolonged time (21 h, Table S6, entry 2). When I_2_ was used in catalytic amounts, Au was dissolved quantitatively (Table 1, entry 1). Noteworthy, when either 2‐MBI or H_2_O_2_ was excluded from the reaction, only minor dissolution is observed (Table 1, entries 3 and 4). The sum of dissolution percentages of cases presented in Entries 2, 3, and 4 is therefore less than that shown in Entry 1, which is a main proposition for plausible catalytic cycle.

**Table 1:** Indication of cooperation between ligand, oxidant and catalyst.^[a]^

Entry	2‐MBI [equiv]	H_2_O_2_ [equiv]	I_2_ [equiv]	Au dissolved [%]^[b]^
1	20	20	0.1	100
2	20	20	–	48
3	20	–	0.1	7
4	–	20	0.1	2

[a] Reaction parameters (when applicable): Au powder (2 mg, 0.01 mmol), 20 equiv of 2‐MBI (0.2 mmol, 30 mg), EtOH (10 mL), 0.1 equiv of I_2_ (0.001 mmol, 0.254 mg), 20 equiv of H_2_O_2_ (33 % aq., 0.2 mmol), 60 °C, 13 h. [b] Determined by flame atomic absorption spectroscopy (FAAS).

**Figure 2:**
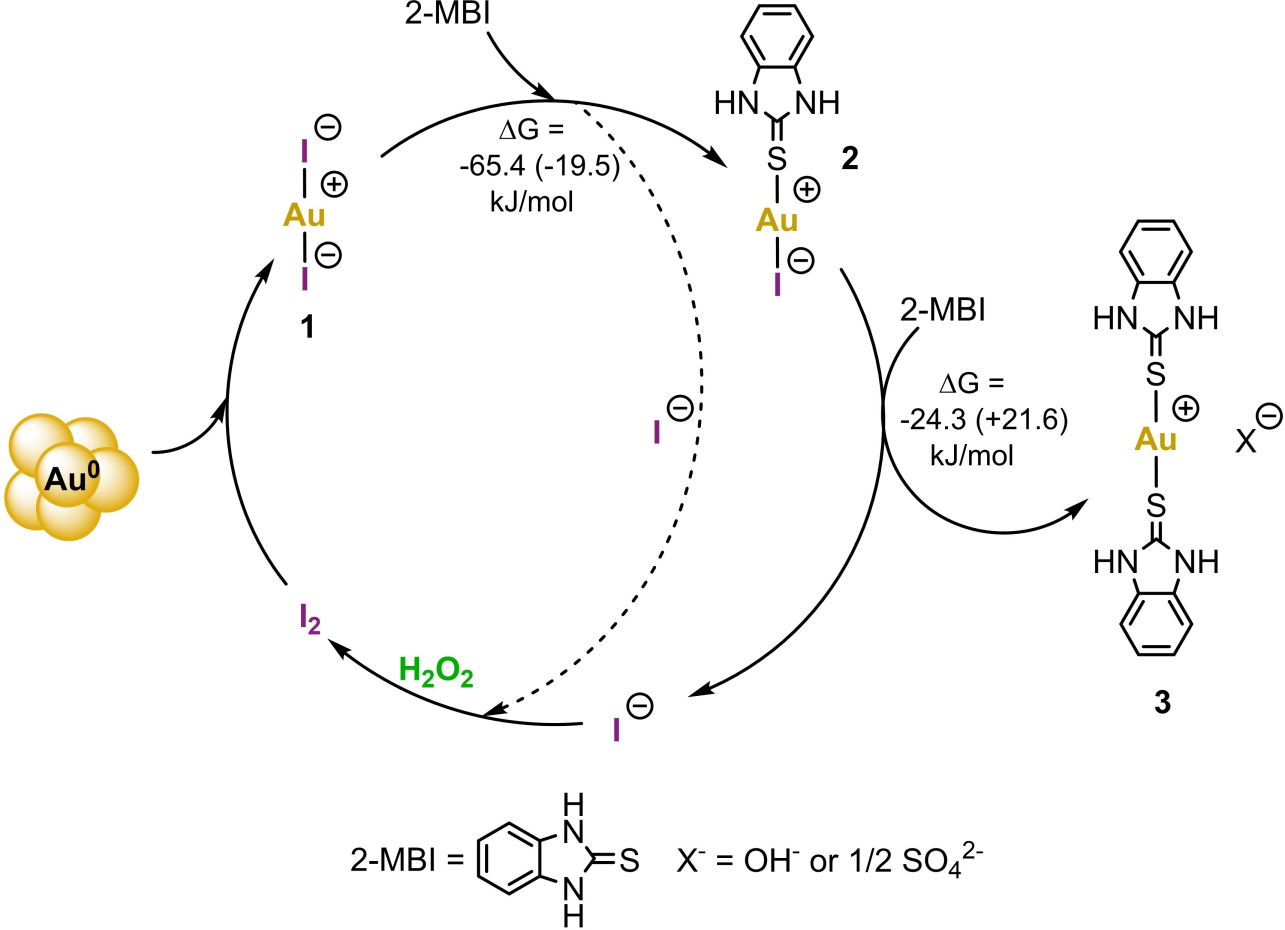
Mechanism of I_2_‐catalysed Au dissolution. Elemental Au is oxidized by I_2_ and dissolves in EtOH as **1** (identified form ESI‐HRMS negative mode as species with *m*/*z* 450.7750). After, **1** undergoes a substitution reaction on Au^I^ centre where one iodide ligand is exchanged with one 2‐MBI molecule and formed species **2** is detected as [*M*−H]^−^ with *m*/*z* 472.8870. Free iodide(s) is re‐oxidized by H_2_O_2_ to generate I_2_. The second iodide in **2** is again replaced with yet another 2‐MBI molecule and stable species **3** is produced and identified from ESI‐HRMS spectra in positive and negative mode as [*M*]^+^ (*m*/*z* 497.0162) and [*M*−2 H]^−^ (*m*/*z* 494.9998), respectively. The second iodide is again regenerated by H_2_O_2_ and formed I_2_ can return to the start of the catalytic cycle. The change of Gibbs free energy (Δ*G*) for the substitution reaction of first and second iodide was calculated for high (first value) and low concentration (value in brackets) of I_2_ at TPSS‐D3/def2‐TZVP level. OH^−^ or SO_4_^2−^ can serve as a counterion for **3**.

**Figure 3:**
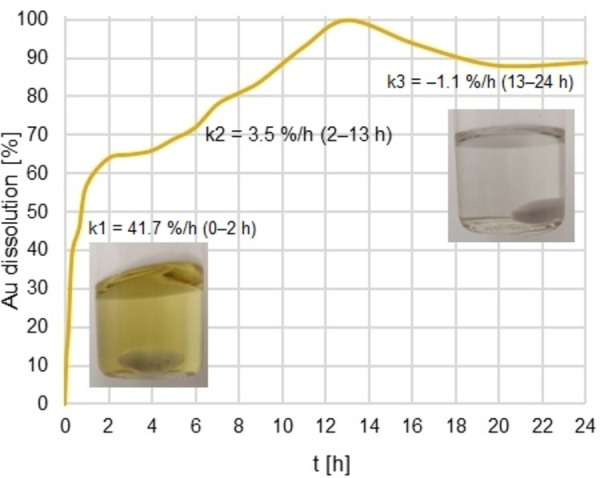
Au dissolution curve in 24 h. Dissolution process was monitored by FAAS for 24 h and dissolution curve was plotted against time. *k*_1_, *k*_2_, and *k*_3_ are predominant dissolution rates at different times of the reaction progress (0–2 h, 2–13 h and 13–24 h, respectively). They were calculated as tangential rates with slopes of 41.7 % h^−1^, 3.5 % h^−1^, and −1.1 % h^−1^, respectively. I_2_ concentration drop is manifested as a discoloration of the reaction after 2 h.

Dissolution of Au proceeds through several mechanistic steps. First, I_2_ oxidizes Au^0^ to Au^I^ by forming [AuI_2_]^−^ species (Figure 2, **1**) identified from the ESI‐HRMS spectra (Figure S8). **1**, a labile complex, readily undergoes a ligand exchange reaction, and one iodide ligand is substituted with a *S‐*bonding 2‐MBI molecule in its dominant thione tautomeric form.[^54^, ^59^, ^60^, ^61^] The formation of a neutral mixed‐ligand species (Figure 2, **2**) is a fingerprint of ligand exchange reaction and observed by ESI‐HRMS as [*M*−H]^−^ (Figure S9). After the second iodide is replaced with yet another 2‐MBI molecule, relatively stable complex **3**[^59^, ^60^, ^61^] (Figure 2, **3**) is formed and detected by ESI‐HRMS in positive and negative mode as [*M*]^+^ (Figure S13) and [*M*−2 H]^−^ (Figure S10), respectively. Both, OH^−^ or SO_4_^2−^ that are produced by redox reactions between H_2_O_2_ and I^−^ or 2‐MBI, could serve as a counterion for **3**.^[54]^ Free iodide ions are then re‐oxidized by H_2_O_2_, and newly formed I_2_ returns to the catalytic cycle (Figure 2). This is supported by earlier reports in organic synthesis[^62^, ^63^] and published redox potential series.^[64]^ Although in situ regeneration of I_2_ has been proposed,[^28^, ^29^, ^30^, ^31^, ^52^] no Au dissolution using only catalytic amounts of I_2_ has been reported.

Dissolution progress was monitored by flame atomic absorption spectroscopy (FAAS) for 24 h and a graph of total dissolved Au vs. time was plotted (Figure 3). Three main reaction rates (*k*_1_, *k*_2_, and *k*_3_) are predominant at different time periods during the 24 h reaction. In the beginning, I_2_ concentration is the highest, causing the steepest reaction slope of rate *k*_1_ (0–2 h, 41.7 %/h), which is also resembled in the brownish colour of the ethanol solution (Figure S4). Even though H_2_O_2_ is already re‐oxidizing iodide to I_2_, it's being consumed by a presumably faster reaction, Au oxidation. This makes I_2_ regeneration the rate limiting factor for Au dissolution. It manifests itself as flatter slope for reaction rate *k*_2_ (2–13 h, 3.5 %/h) and discoloration of the reaction mixture (Figure S4). From 2 h onwards, I_2_ exists in low, yet sufficient, concentration for the catalytic cycle to proceed. However, after approximately 13 h, concentration of the 2‐MBI ligand is depleted due to the redox reaction with H_2_O_2_ (see below) and some precipitation of Au occurs. This is revealed as reaction rate with a flat and negative slope, *k*_3_ (13–24 h, −1.1 %/h). As seen from the dissolution curve (Figure 3), complete dissolution is reached at 13 h. In general, it is worth to notice that dissolution of gold in organic solution is faster than optimized cyanide leaching^[54]^ but it is very much depending on the ligand, ligand concentration and reaction conditions. Here as the reaction is based on just catalytic amount of I_2_, the dissolution rate is lower (0.15 mg h^−1^) than in previous examples with marked excess of 4‐PS^[54]^ (12.9 mg h^−1^) but only slightly lower than in the recently reported bi‐ligand systems^[59]^ (1 mg h^−1^).

**Figure 4:**
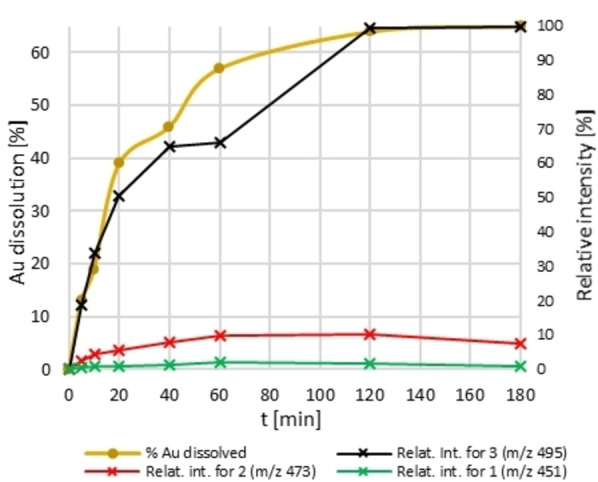
Au dissolution and relative intensities for species **1**, **2**, and **3** in the first 3 h. Intensities for species **1** ([*M*]^−^ with *m*/*z* 451), **2** ([*M*−H]^−^ with *m*/*z* 473), and **3** ([*M*−2 H]^−^ with *m*/*z* 495) were monitored by ESI‐HRMS in negative mode for the first three hours of the dissolution reaction. The highest intensity was set to 100 % and other data was adjusted accordingly. Intensities were combined with FAAS dissolution curve and plotted against time. Intensities of **1** and **2** rise at the beginning and are then constantly present to some extent, while the intensity for **3** closely follows the dissolution curve (which is in agreement with the proposed mechanism). At 3 h, the ratio between **1**, **2**, and **3** reaches 1.1/7.7/100.0.

Since the main change in the reaction speed, as well as the discolouration, occurs after 2 h, we monitored the concentrations of Au species **1**, **2**, and **3** in the first three hours of the reaction. The intensities of different reaction species were plotted against time using the data acquired from ESI‐HRMS negative mode. The total amount of dissolved Au measured with FAAS was added for comparison (Figure 4). The intensity curve for **3** closely follows the dissolution curve and remains the main Au species throughout the whole dissolution process. Rapid ligand exchange reaction is noted as peaks for **2** and **3** emerge only after 5 min. According to the mechanism, concentrations of **1** and **2** rise at the beginning and are both then constantly present in the reaction as minor Au species with a ratio of 1.1(**1**)/7.7(**2**)/100.0(**3**) even at 3 h.

We performed DFT calculations related to the geometry and stability of the above‐described intermediates to gain further insight into the process at the TPSS‐D3/def2‐TZVP/CPCM (ethanol) level. The change of Gibbs free energy (Δ*G*) for substitution reactions from **1** to **2** and from **2** to **3** were determined as the difference between Gibbs energies of products and reactants (Figure S30). When I^−^ and I_2_ are both present in the solution, I_3_^−^ is readily formed with a large stability constant in ethanol,^[65]^ which was considered when calculating Δ*G*. The first substitution reaction, in which either iodide of complex **1** is replaced by 2‐MBI, is a thermodynamically favourable reaction regardless of I_2_ concentration; At high concentrations resembling the initial state of the reaction, Δ*G* is −65.4 kJ mol^−1^ and at low I_2_ concentrations resembling the end state of the reaction, −19.5 kJ mol^−1^, respectively. The spontaneity for substitution of the second iodide relies on I_2_ concentration. With higher concentrations of I_2_, formation of **3** is spontaneous (−24.3 kJ mol^−1^) but becomes thermodynamically less favourable when I_2_ concentrations are depleted (+21.6 kJ mol^−1^) as in the end of the reaction. This is in correlation with the observed lower reaction rate after 3 h and the disappearance of the brownish iodine colour. Small positive Δ*G* for overall substitution (+2.1 kJ mol^−1^) therefore explains why 20 equivalents of 2‐MBI are still needed to push the equilibrium towards **3** and free iodide ions after I_2_ concentrations are depleted.

**Figure 5:**
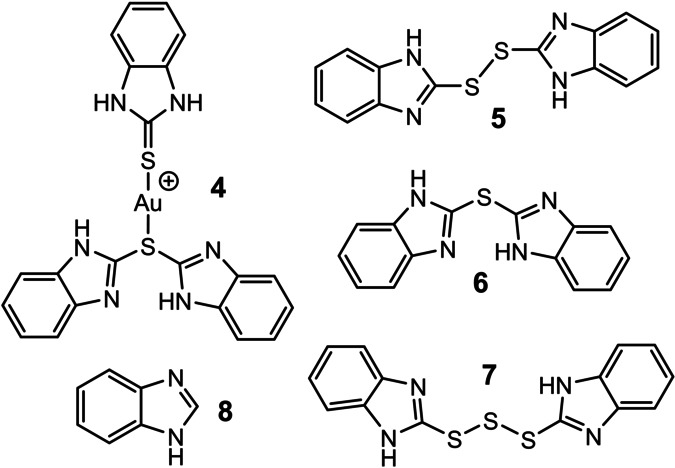
2‐MBI derivatives and another Au species identified in the reaction mixture. Different species were identified from ESI‐HRMS in positive mode and ^1^H NMR spectrum of the reaction mixture, including disulphide **5**, sulphide **6**, and trisulphide **7**. Benzimidazole (**8**) and another Au species, (**4**) were also detected by ESI‐HRMS but with low intensity.

During the reaction, 2‐MBI reacts with H_2_O_2_ and several ligand derivatives were identified from ESI‐HRMS and NMR spectra including disulphide **5**, sulphide **6**, and trisulphide **7** (Figure 5). Formation of **5** at the beginning of the reaction course is a result of simple thiol‐to‐disulphide oxidation of 2‐MBI with H_2_O_2_. In addition, 2‐MBI or **5** has to undergo a disproportionation reaction to generate **6** and **7** simultaneously after 1 h. After 3 h, ESI‐HRMS studies revealed another mixed‐ligand Au^I^ species (Figure 5, **4**) containing **6** and one 2‐MBI molecule. Its peak emerged with low intensity compared to **3**, having a ratio of 0.2(**4**)/100.0(**3**) at 13 h. Desulfurization product **8** was also confirmed by ESI‐HRMS, but its concentration in the reaction is likely low since it was not observed by ^1^H NMR. Similarly, Au complexes **2** and **4**, as well as a main Au species **3**, were not confirmed by ^1^H NMR likely due to their low concentrations, similar proton shifts compared to the free ligands,[^54^, ^59^] and/or fast ligand exchange reaction. Degradation upon NMR sample preparation also cannot be excluded. Even though 2‐MBI is clearly reacting in these oxidative conditions, it is still irreplaceable as a strongly coordinating ligand capable of substituting iodide on a Au^I^ centre and with this reducing amount of iodine needed for complete dissolution of gold.

**Figure 6:**
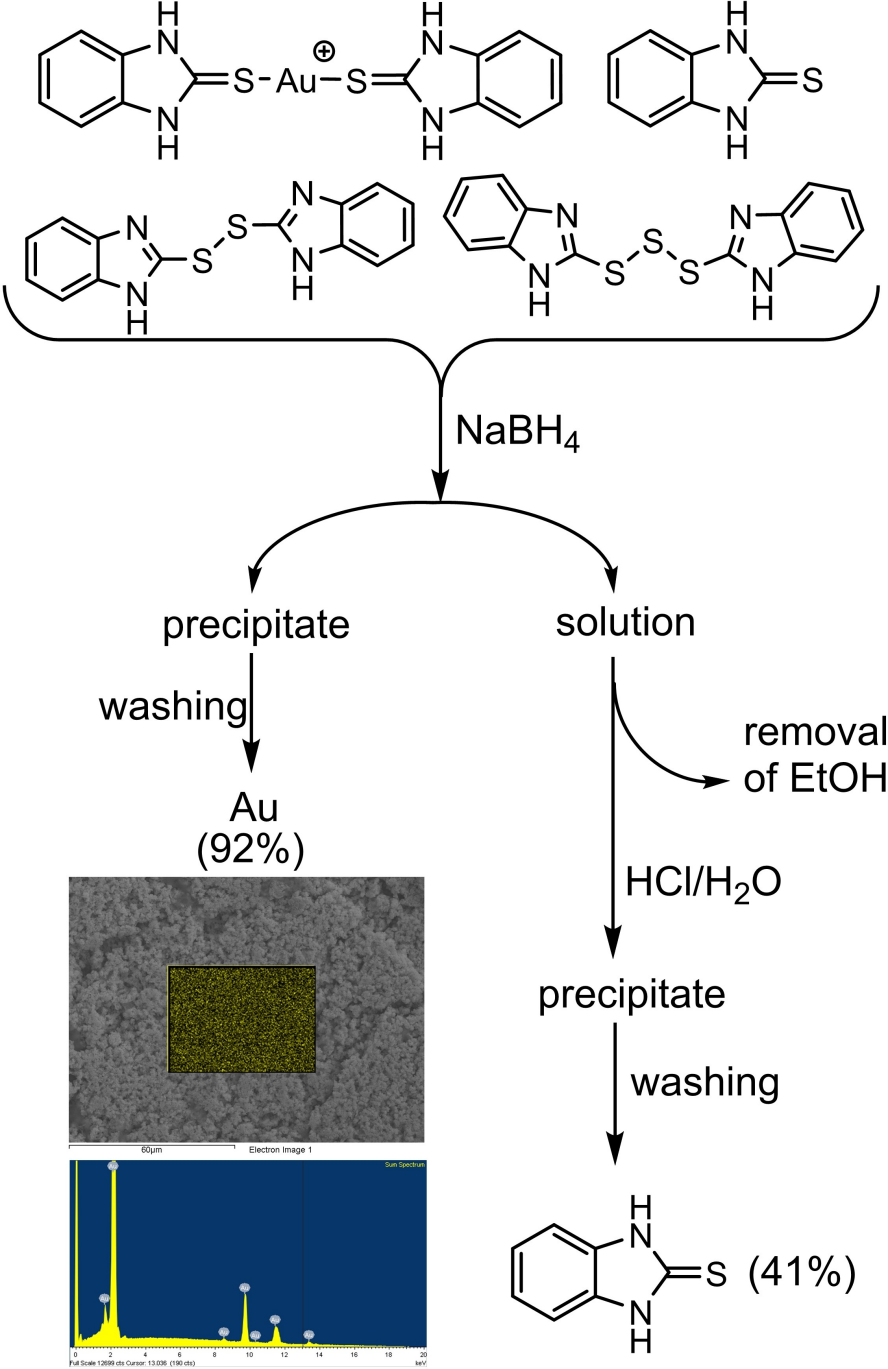
Precipitation of Au and recycling of 2‐MBI with NaBH_4_. NaBH_4_ was added directly to the scaled‐up reaction mixture with 20 mg of dissolved Au. Black quantitative precipitate was collected and identified with SEM and EDS as elemental Au with 92 % isolated yield. After evaporation of reaction solvent and quenching of the residue with diluted aqueous HCl, pure 2‐MBI was regenerated with 41 % yield.

Next, we investigated ways to close the Au recycling loop. NaBH_4_ was chosen as an example of the reducing agent capable in transforming disulphides to thiols^[66]^ and Au ions to their elemental form,^[3]^ and therefore offering possibility for one‐step recycling process (Figure 6). Reduction was demonstrated by adding NaBH_4_ directly into the scaled‐up reaction mixture with 20 mg of dissolved Au, resulting in formation of a black precipitate which was collected, thoroughly washed, and dried. A sample was taken and analysed using scanning electron microscopy (SEM) and energy‐dispersive X‐ray spectroscopy (EDS) to conclude that it was in fact elemental Au with a particle size of 10–20 nm, acquired as a quantitative precipitate with 92 % yield. By evaporation of reaction solvent and subsequent quenching of the residual NaBH_4_ with diluted aqueous HCl, pure 2‐MBI in 41 % yield was collected as precipitate. Reduction of disulphide **5** and trisulfide **7** to 2‐MBI is evident from crude ^1^H NMR spectra of the reaction mixture before and after reduction (Figures S27–S29). Quantitative precipitation of Au and partial reduction of side reaction products back to 2‐MBI increases the sustainability of the recycling process.

## Conclusion

In conclusion, we developed a new strategy for the sustainable gold dissolution using catalytic amounts (10 mol %) of I_2_ and ethanol as green solvent. Au was successfully dissolved with mild heating (60 °C) using 20 equivalents of inexpensive, safe, and partially recyclable ligand, 2‐MBI, and 20 equivalents of environmentally benign oxidant, aqueous H_2_O_2_. 100 % dissolution was reached after 13 h with **3** being the main Au species as proven by ESI‐HRMS. The I_2_ regenerative cycle for Au dissolution proceeds through four mechanistic steps: oxidations of Au with elemental I_2_ and dissolution of formed **1**, subsequent substitution of both iodides on Au^I^ centre with two 2‐MBI molecules producing **2** and then **3**. This is followed by re‐oxidation of free iodide ions by H_2_O_2_ to form I_2_, which returns back to the catalytic cycle. The mechanism was supported by detecting important species with ESI‐HRMS and measuring ratios between their intensities through time and by DFT calculations. To close the recycling loop, we demonstrated the quantitative precipitation of Au and recycling of 2‐MBI in a one‐step procedure using NaBH_4_ as a reductant. With their high demand and limited sources, recycling of precious metals like Au will become unavoidable in the foreseeable future. Since environmental impact is often ignored, solving this issue by employment of established methods creates another troubling problem of polluting Earth's crust with large quantities of toxic waste. With this in mind, dissolving Au under very mild conditions with a safe, inexpensive, and environmentally benign mixture of now daily used disinfectants like ethanol, hydrogen peroxide, and iodine tincture seems very promising. The astonishing fact is that gold can be dissolved in ethanol with just catalytic amounts of iodine. To further improve the sustainability of this system a logical future direction is to find a ligand with high complex constant and oxidative stress resistance allowing its stoichiometric use in atom‐economic dissolution. We also anticipate that a similar process can be applied in the dissolution of other precious metals by appropriate choice of ligand.

## Conflict of interest

The authors declare no conflict of interest.

1

## Supporting information

As a service to our authors and readers, this journal provides supporting information supplied by the authors. Such materials are peer reviewed and may be re‐organized for online delivery, but are not copy‐edited or typeset. Technical support issues arising from supporting information (other than missing files) should be addressed to the authors.

Supporting Information

## Data Availability

The data that support the findings of this study are available in the Supporting Information of this article.
