# Supplementary material for: Iodine‐Catalysed Dissolution of Elemental Gold in Ethanol
Source: Angew Chem Int Ed Engl. 2022 Feb 15;61(14):e202117587. doi: 10.1002/anie.202117587 (PMC9305299; doi:10.1002/anie.202117587)
Supplement: Supplementary file 1 — Supporting Information [file ANIE-61-0-s001.pdf]

Angewandte Chemie
Eine Zeitschrift der Gesellschaft Deutscher Chemiker

Supporting Information

Iodine-Catalysed Dissolution of Elemental Gold in Ethanol

*A. Zupanc, E. Heliövaara, K. Moslova, A. Eronen, M. Kemell, Č. Podlipnik, M. Jereb, T. Repo**

Table of Contents

1. Materials and methods ... 3
2. Au dissolution procedures ... 3
2.1. Optimized procedure for Au dissolution ... 3
2.2. Scale-up procedure for Au dissolution ... 3
3. FAAS measurement ... 3
3.1. Optimization of reaction parameters ... 4
3.2. Au dissolution vs. time ... 7
4. Colour of the reaction mixture ... 9
5. ESI-HRMS studies ... 9
5.1. Sample preparation ... 9
5.2. Found species ... 10
5.2.1. Negative ion mode ... 10
5.2.2. Positive ion mode ... 12
5.3. Intensities for selected species vs. time ... 16
6. Recycling of Au and 2-MBI ... 16
6.1. Procedure ... 16
6.2. FESEM-EDS analysis ... 18
6.3. NMR study of reaction mixture before and after reduction ... 18
7. Computational details ... 20
7.1 Calculation of ΔG for substitution reactions ... 20
7.2 Calculated energies and cartesian coordinates for different species ... 21
References ... 27
Author Contributions ... 27

1. Materials and methods

All chemicals were bought from commercial sources and used without further purification. Au powder (1.5-3.0 μm spherical, 99.9 %) was purchased from Strem Chemicals, 2-mercaptobenzimidazole (2-MBI), 4-pyridinethiol (4-PS), $NaBH_4$, $K(AuCl_4)$ (98%) and iodine from Sigma Aldrich and 33% aqueous H_2O_2 from VWR International. All solvents were HPLC grade.
Flame atomic absorption spectroscopy (FAAS) measurements were performed on a Perkin-Elmer 3030 atomic absorption spectrophotometer. Measurements were carried out in air/acetylene flame and by using Au hollow cathode lamp (HCL) at wavelength of 248 nm with lamp current of 10 mA.
High-resolution electrospray-ionization mass spectra (ESI-HRMS) were recorded with a Bruker microTOF mass spectrometer in a positive and negative ion mode using sodium formate as a calibrant.
An Oxford INCA 350 energy-dispersive X-ray microanalysis system connected with a Hitachi S-4800 field emission scanning electron microscope (FESEM) was used for the energy-dispersive X-ray spectrometry (EDS) measurements.
All NMR spectra were recorded in DMSO-*d*$_6$ with a Varian Mercury 400 instrument (at 400 MHz) using Me_4Si as an internal standard. Chemical shifts are reported in ppm (δ) relative to central lines of DMSO-*d*$_6$ for ^{1}H NMR (δ = 2.50 ppm) and ^{13}C NMR (δ = 39.52 ppm).

2. Au dissolution procedures

2.1. Optimized procedure for Au dissolution

Au powder (2 mg, 0.01 mmol, 1.5-3.0 μm spherical particles) and 2-MBI (20 eq., 30 mg, 0.2 mmol) were weighted into a 25 mL glass vial equipped with oval magnetic stirring bar. 10 mL of EtOH were added and the mixture was stirred until all 2-MBI was dissolved. Then the reaction was charged with 51.2 μL of freshly prepared 19.5 mM EtOH solution of I_2 (10 mol %, 0.254 mg, 0.001 mmol) and 33% aqueous H_2O_2 (20 eq., 19 μL, 0.2 mmol). Reaction vial was tightly closed with a plastic cap, stirred, and submerged into a preheated oil bath at 60 °C. After 13 h of vigorous stirring, 100% dissolution of Au was reached.

2.2. Scale-up procedure for Au dissolution

Au powder (20 mg, 0.1 mmol, 1.5-3.0 μm spherical particles) and 2-MBI (20 eq., 300.4 mg, 2 mmol) were weighted into a 250 mL round bottom flask equipped with magnetic stirring bar. The majority of EtOH (100 mL in total) was added into the flask to dissolve 2-MBI. Then, I_2 (10 mol %, 2.5 mg, 0.01 mmol) was dissolved in residual EtOH and added to the reaction mixture followed by addition of 33% aqueous H_2O_2 (20 eq., 186 μL, 2 mmol). Flask was tightly sealed with a plastic cap, stirred and submerged into a preheated oil bath at 60 °C. All Au was dissolved after 13 h of vigorous stirring.

3. FAAS measurement

The amount of dissolved Au was determined by flame atomic absorption spectroscopy (FAAS) measurements that were performed with a Perkin-Elmer 3030 atomic absorption spectrophotometer. Measurements were carried out in air/acetylene flame and by using Au hollow cathode lamp (HCL) at wavelength of 248 nm with lamp current of 10 mA.
Calibration curve (Figure S1) was prepared from stock solution of potassium tetrachloroaurate(III) in EtOH with concentrations of 2, 4, 6, 8 and 10 mg/L. Sample was taken from reaction solution (300 μL) and diluted with EtOH (9.5 mL, total sample volume 9.8 mL). Each dissolution experiment was repeated more than once to ensure the dissolution consistency. To minimize systematic error of sample preparation, reference calibration curves and reference samples were measured periodically.

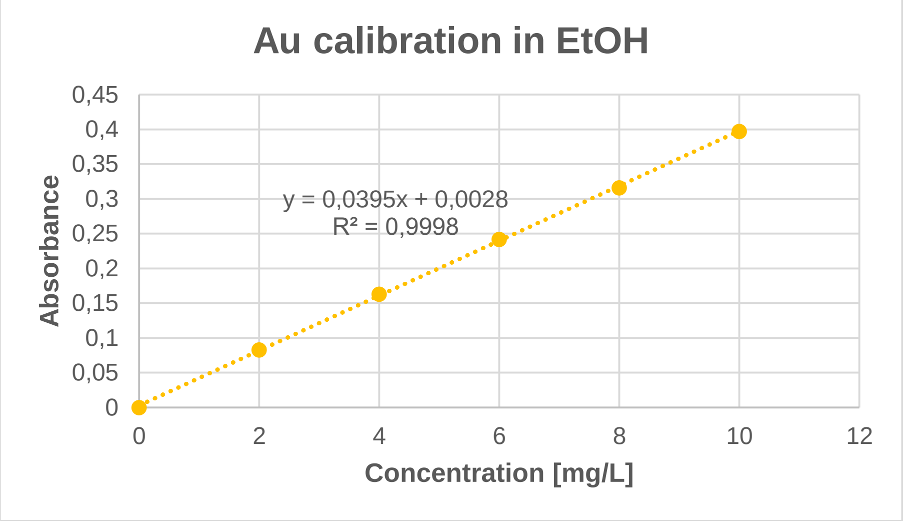

Figure S1: Au calibration curve in EtOH. Standards were prepared in EtOH in the following Au concentrations: 2, 4, 6, 8 and 10 mg/L.

3.1. Optimization of reaction parameters

Table S1 summarizes the optimization results for 4-PS assisted dissolution of Au, including the amount of 4-PS, H_2O_2, and I_2, solvent and the reaction temperature. Reaction setup and the order of reagent addition was similar to the procedure with optimized conditions described above (Section 2.1.). As seen from Table S1, 4-PS is unable to substitute iodide in formed $[AuI_2]^-$, since the sum of dissolved Au (%) in experiments excluding H_2O_2 (entries 2, 4, 7 and 10) or I_2 (entries 1, 6 and 9) is the same or more than in experiment with all three components, ligand, oxidant and catalyst, respectively (entries 3, 5, 8 and 11).

Table S1: Dissolution of Au with 4-PS. Quantities are calculated according to Au powder (2 mg, 0.01 mmol).

Entry	4-PS [eq]	H_2O_2[a] [eq]	I_2 [mol %]	Solvent (5 mL)	T [°C]	% Au diss. (1 h)[b]	% Au diss. (2 h)[b]	% Au diss. (21 h)[b]	% Au diss. (23 h)[b]
1	10	10	0	DMF	rt	ND[c]	5	ND[c]	44
2	10	0	25	DMF	rt	ND[c]	49	ND[c]	54
3	10	10	25	DMF	rt	ND[c]	54	ND[c]	72
4	10	0	12.5	DMF	rt	ND[c]	26	ND[c]	32
5	10	10	12.5	DMF	rt	ND[c]	29	ND[c]	53
6	10	10	0	DMF	60	ND[c]	48	ND[c]	ND[c]
7	10	0	12.5	DMF	60	ND[c]	25	ND[c]	ND[c]
8	10	10	12.5	DMF	60	ND[c]	57	ND[c]	ND[c]
9	10	10	0	EtOH	rt	2	ND[c]	1	ND[c]
10	10	0	12.5	EtOH	rt	15	ND[c]	18	ND[c]
11	10	10	12.5	EtOH	rt	16	ND[c]	8	ND[c]

[a]33% aq. [b]Determined by FAAS. [c]Not determined.

Similarly, Table S2 shows results for 2-MBI assisted dissolution of Au in DMF, including the amount of 2-MBI, H_2O_2, and I_2, and the reaction temperature. Reaction setup and the order of reagent addition was similar to the procedure with optimized conditions described above (Section 2.2). As seen from Table S2, 2-MBI can substitute iodide in formed $[AuI_2]^-$, since the sum of dissolved Au (%) in experiments excluding H_2O_2 (entries 2, 5 and 8) or I_2 (entries 1, 4 and 7) is less than in experiment with all three components at any time point at 60 °C or at room temperature (entries 3, 6 and 9). Cooperation between 2-MBI, H_2O_2 and I_2 is therefore proposed.

Table S2: Dissolution of Au with 2-MBI in DMF (5 mL). Quantities are calculated according to Au powder (2 mg, 0.01 mmol).

Entry	2-MBI [eq]	H_2O_2[a] [eq]	I_2 [mol%]	T [°C]	% Au diss. (2 h)[b]	% Au diss. (18 h)[b]	% Au diss. (22 h)[b]
1	20	20	0	rt	ND[c]	0	ND[c]
2	20	0	12.5	rt	ND[c]	15	ND[c]
3	20	20	12.5	rt	ND[c]	20	ND[c]
4	20	20	0	60	2	14	15
5	20	0	12.5	60	15	13	17
6	20	20	12.5	60	30	66	74
7	40	40	0	60	8	ND[c]	33
8	40	0	12.5	60	15	ND[c]	17
9	40	40	12.5	60	42	ND[c]	89

[a]33% aq. [b]Determined by FAAS. [c]Not determined.

Other solvents than DMF were tested as potential reaction media for Au dissolution (Table S3). In some solvents, a precipitation was formed, which made it impossible to detect Au in solution with FAAS. The precipitation was also formed in EtOH but became soluble in larger amounts of the solvent. Therefore, dissolution in EtOH was further studied (Table S4). As Table S4 shows, precipitation formed when using 5 mL of EtOH even at room temperature (Table S4, entry 2) or with lower amount of ligand at elevated temperature (Table S4, entry 3). Yet, near quantitative dissolution of Au was achieved in 10 mL of EtOH (Table S4, entry 4).

Table S3: Solvent screening for Au dissolution*.

Solvent (5 mL)	% Au diss. (2 h)[a]	% Au diss. (22 h)[a]
EtOH	N/A[b]	N/A[b]
EtOAc	0	7
MeCN	1	21
AcOH	N/A[b]	N/A[b]
THF	N/A[b]	N/A[b]
ethylene glycol	36	9
diglyme	N/A[b]	N/A[b]
H_2O	N/A[b]	N/A[b]

*Reaction conditions: Au (2 mg, 0.01 mmol), 2-MBI (20 eq.), 33% aq. H_2O_2 (20 eq.), I_2 (12.5 mol %), solvent (5 mL), 60°C. [a]Determined by FAAS. [b]Not available as precipitation forms during dissolution.

Table S4: Preliminary screening of reaction parameters for Au dissolution with 2-MBI in EtOH. Quantities are calculated according to Au powder (2 mg, 0.01 mmol).

Entry	2-MBI [eq]	H_2O_2[a] [eq]	I_2 [mol %]	EtOH [mL]	T [°C]	% Au diss. (2.5 h)[b]	% Au diss. (24 h)[b]
1	20	20	12.5	5	60	N/A[c]	N/A[c]
2	20	20	12.5	5	rt	N/A[c]	N/A[c]
3	10	10	12.5	5	60	N/A[c]	N/A[c]
4	20	20	12.5	10	60	73	91

[a]33% aq. [b]Determined by FAAS. [c]Not available as precipitation forms during dissolution.

In Table S5, a drop in dissolution efficiency is detected when 96% EtOH is used instead of pure EtOH. Cooperation between 2-MBI, H_2O_2 and I_2 is suppressed since the sum of Au dissolution percentages in experiments excluding I_2 (Table S5, entry 2), H_2O_2 (Table S5, entry 3) and 2-MBI (Table S5, entry 4) is more than in experiment with all three components (Table S5, entry 1).

Table S5: Dissolution of Au in 96% EtOH. Quantities are calculated according to Au powder (2 mg, 0.01 mmol) of Au.

Entry	2-MBI [eq]	H_2O_2[a] [eq]	I_2 [mol %]	96% EtOH [mL]	T [°C]	% Au diss. (21 h)[b]
1	20	20	12.5	10	60	56
2	20	20	0	10	60	25
3	20	0	12.5	10	60	27
4	0	20	12.5	10	60	3
5	20	20	10	10	60	67
6	20	40	10	10	60	43
7	30	30	6.25	15	60	69
8	20	20	12.5	10	rt	63
9	30	30	12.5	15	rt	75
10	20	40	10	10	rt	49

[a]33% aq. [b]Determined by FAAS.

Table S6 summarizes the final optimization of the reaction parameters using pure EtOH. Dissolution condition with adequately minimal amounts of reagents with acceptable dissolution efficiency was chosen for further studies (Table S6, entry 1). As it was proven later, 100% dissolution was achieved after 13 h (Table S8 and Figure S2). The sum of Au dissolution percentages in experiments excluding I_2 (Table S6, entry 2 or Table S7, entry 2), H_2O_2 (Table S6, entry 3 or Table S7, entry 3) and 2-MBI (Table S6, entry 4 or Table S7, entry 4) is less than in experiment with all three components (Table S6, entry 1 or Table S7, entry 1) at 21 h (Table S6) or at 13 h (Table S7) what is a proof of cooperation between all three reagents.

Table S6: Optimization of reaction parameters for Au dissolution in EtOH. Quantities are calculated according to Au powder (2 mg, 0.01 mmol).

Entry	2-MBI [eq]	H_2O_2[a] [eq]	I_2 [mol %]	EtOH [mL]	T [°C]	% Au diss. [19 h][b]	% Au diss. [21 h][b]
1	20	20	10	10	60	89	88
2	20	20	0	10	60	ND[d]	48
3	20	0	10	10	60	ND[d]	4
4	0	20	10	10	60	ND[d]	2
5	30	30	10	15	60	ND[d]	89
6	30	20	10	15	60	ND[d]	94
7	30	30	5	15	60	ND[d]	93
8	15	15	10	10	60	ND[d]	67
9	10	10	5	5	60	55	ND[d]
10	20	20	5	10	60	81	ND[d]
11	10	10	10	10	60	52	ND[d]
12	20	20	10	10	rt	ND[d]	N/A[c]
13	30	30	10	15	rt	ND[d]	N/A[c]
14	30	30	5	15	rt	ND[d]	88
15	35	35	10	20	rt	ND[d]	73
16	40	40	10	20	rt	ND[d]	84
17	20	20	5	10	rt	N/A[c]	ND[d]
18	20	20	5	15	rt	53	ND[d]
19	20	20	10	15	rt	41	ND[d]

[a]33% aq. [b]Determined by FAAS. [c]Not available as precipitation forms during dissolution. [d]Not determined.

Table S7: Proof of cooperation between 2-MBI, H_2O_2 and I_2 in dissolution of Au in EtOH (10 mL) at 60 °C in 13 h. Quantities are calculated according to Au powder (2 mg, 0.01 mmol).

2-MBI [eq]	H_2O_2[a] [eq]	I_2 [mol %]	% Au diss. (13 h)[b]
20	20	10	100
20	20	0	48
20	0	10	7
0	20	10	2

[a]33% aq. [b]Determined by FAAS.

3.2. Au dissolution vs. time

20 samples were taken between 5-180 minutes in the course of 24 h dissolution reaction. Maximum two samples were taken from each reaction mixture to minimize the error arising from reducing the volume. Dissolved Au vs. time graph was constructed from the acquired data (Table S8). As seen from Table S8 and Figure S2, 100% dissolution was achieved after 13 h.

Table S8: Au dissolution vs. time in 24 h reaction.

t [h]	t [min]	Au diss. [%][a]
0	0	0
0.083333	5	13
0.166667	10	19
0.333333	20	39
0.666667	40	46
1	60	57
2	120	64
3	180	65
4	240	66
5	300	69
6	360	72
7	420	78
8	480	81
9	540	84
11	660	93
13	780	100
16	960	94
19	1140	89
21	1260	88
24	1440	89

Reaction conditions: Au powder (2 mg, 0.01 mmol), 2-MBI (30 mg, 0.2 mmol, 20 eq.), EtOH (10 mL), 10 mol % I_2 (0.254 mg, 0.001 mmol), 33% aq. H_2O_2 (19 µL, 0.2 mmol, 20 eq.) at 60 °C. [a]Determined by FAAS.

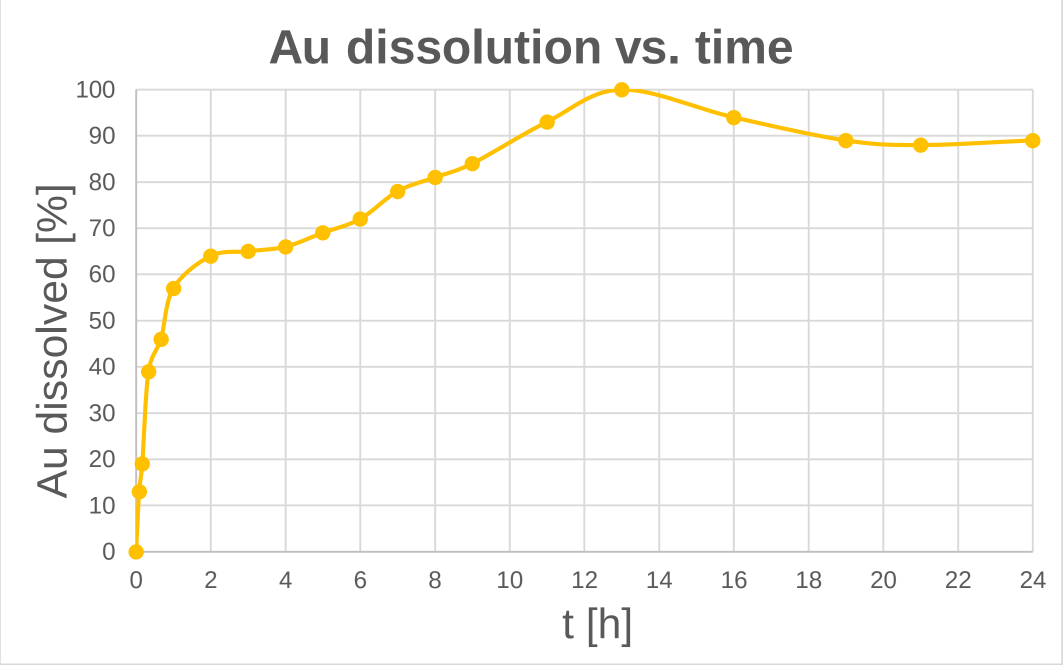

Figure S2: Au dissolution vs. time in 24 h reaction. Reaction conditions: Au powder (2 mg, 0.01 mmol), 2-MBI (30 mg, 0.2 mmol, 20 eq.), EtOH (10 mL), 10 mol % I_2 (0.254 mg, 0.001 mmol), 33% aq. H_2O_2 (19 µL, 0.2 mmol, 20 eq.) at 60 °C. Dissolution percentage were determined by FAAS.

Three different reaction rates (k1, k2 and k3) were predominant at different time periods of the reaction: k1=41.665 %/h (0-2 h), k2=3.471 %/h (2-13 h) and k3=-1.052 %/h (13-24 h) as shown in Figure S3. The tangential lines were drawn to visualize the observed predominant dissolution rates.

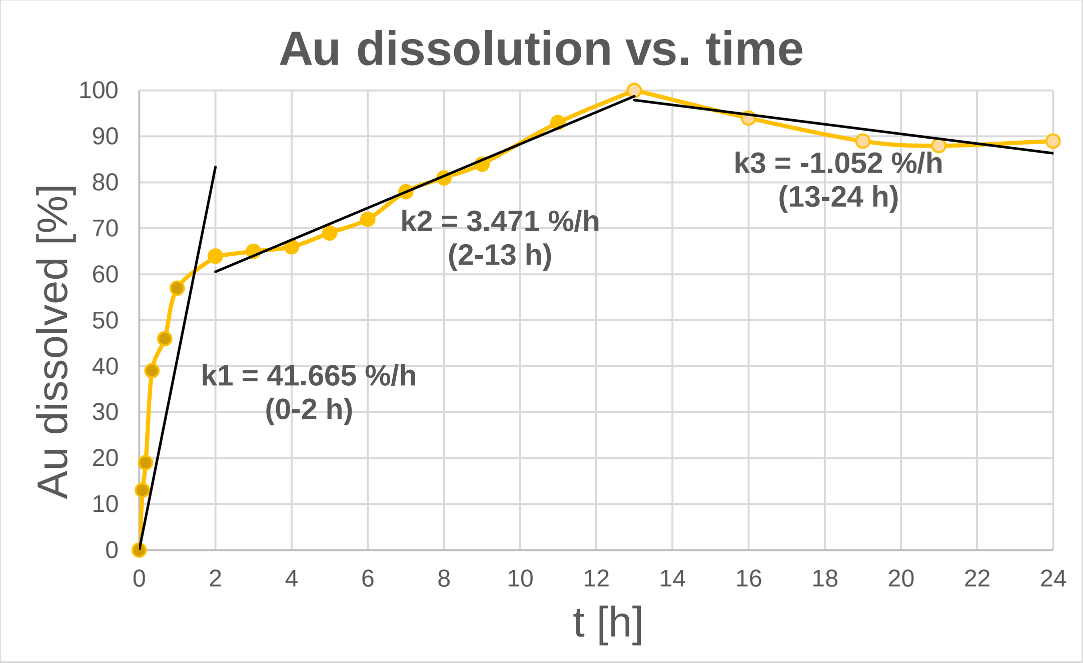

Figure S3: Au dissolution vs. time in 24 h reaction with observed and visualised dissolution rates k1, k2 and k3.

4. Colour of the reaction mixture

Photographs of the reaction were taken according to the dissolution curve (Figure S2) and predominant reaction rates (Figure S3). The photographs in Figure S4 show the colour changes. At the beginning of the reaction (0 min), the solution exhibits brownish iodine colour, which is quickly fading away (30 min) and then completely disappears (2.5 h). Reaction mixture remains colourless till the end of the observation period (24 h). The same applies for a scale-up reaction procedure as seen from photographs represented in Figure S5.

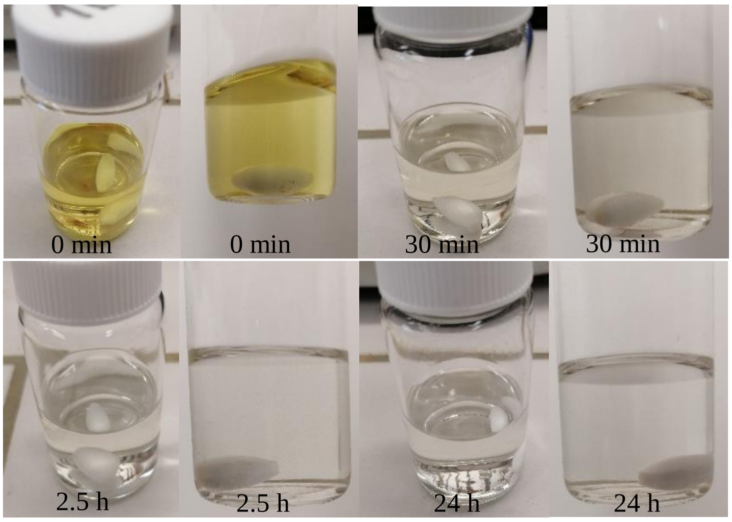

Figure S4: Photographs of the reaction mixture at specific times.

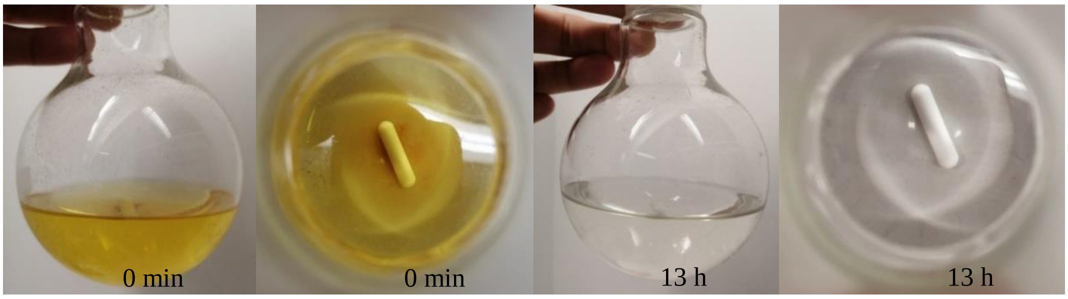

Figure S5: Photographs of a scale-up reaction mixture at 0 min and 13 h.

5. ESI-HRMS studies

5.1. Sample preparation

High-resolution electrospray-ionization mass spectra (ESI-HRMS) were recorded with a Bruker microTOF mass spectrometer in a positive and negative ion mode using sodium formate as a calibrant.
Samples were prepared by taking 20 μL of reaction mixture and diluting with 780 μL of 0.05% aqueous formic acid in MQ water and MeOH mixture (70/30 v/v). Samples were filtered through 0.22 μm PTFE syringe filters prior to measurement. Syringe filters were washed with MeOH before filtration of the sample. Samples were measured immediately to avoid partial degradation and/or precipitation.

5.2. Found species

Experimental isotopic patterns for selected species were compared to calculated patterns. Species found are illustrated in Figure S6.

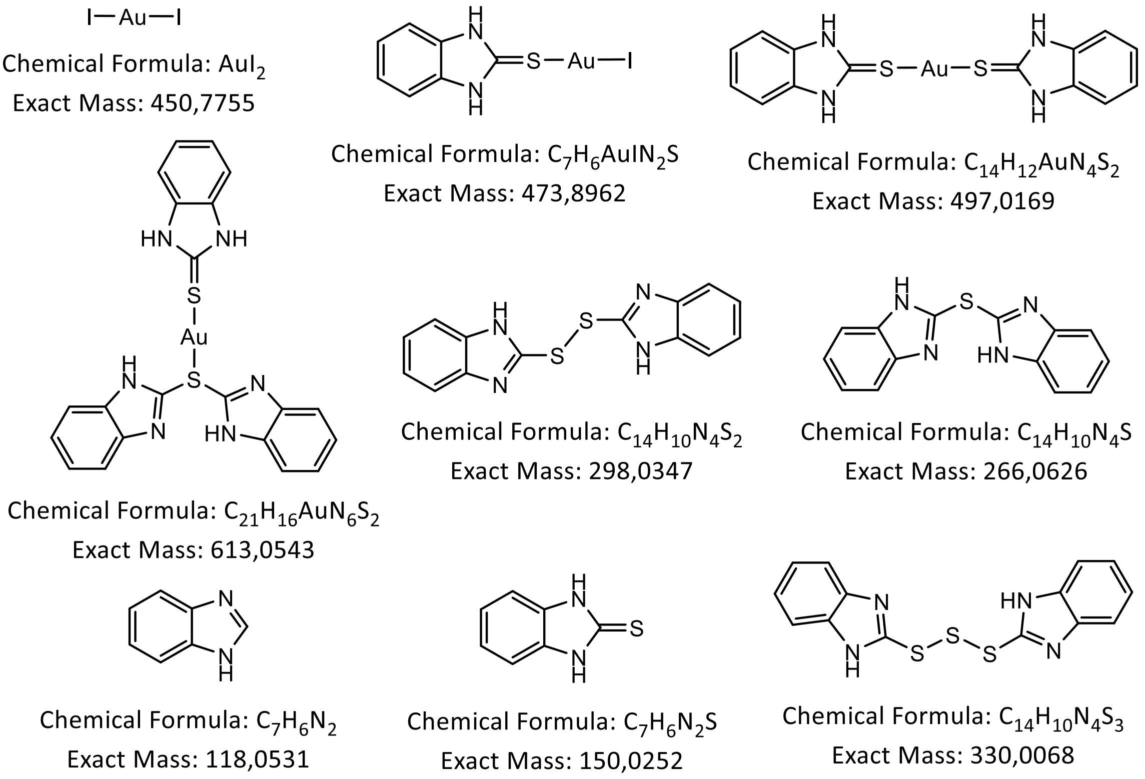

Figure S6: Species found in ESI-HRMS studies.

5.2.1. Negative ion mode

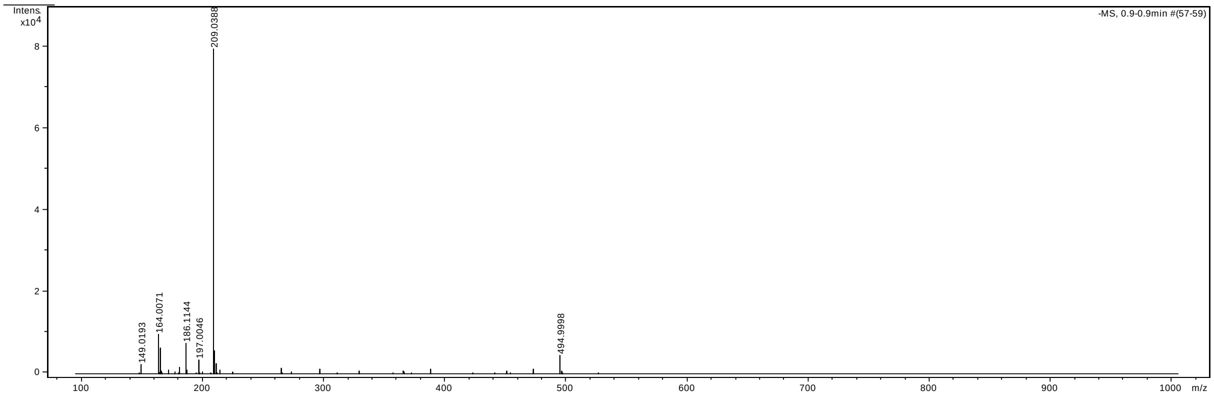

Figure S7: Full range ESI-HRMS spectrum of reaction mixture in negative ion mode. Samples were taken during the standard reaction outlined in section 2.1. Au containing species found: *m/z* 451 $[AuI_2]^-$, *m/z* 473 $[(C_7H_6AuIN_2S)\text{-}H]^-$, *m/z* 495 $[(C_{14}H_{12}AuN_4S_2)\text{-}2H]^-$. Organic species found: *m/z* 297 $[(C_{14}H_{10}N_4S_2)\text{-}H]^-$.

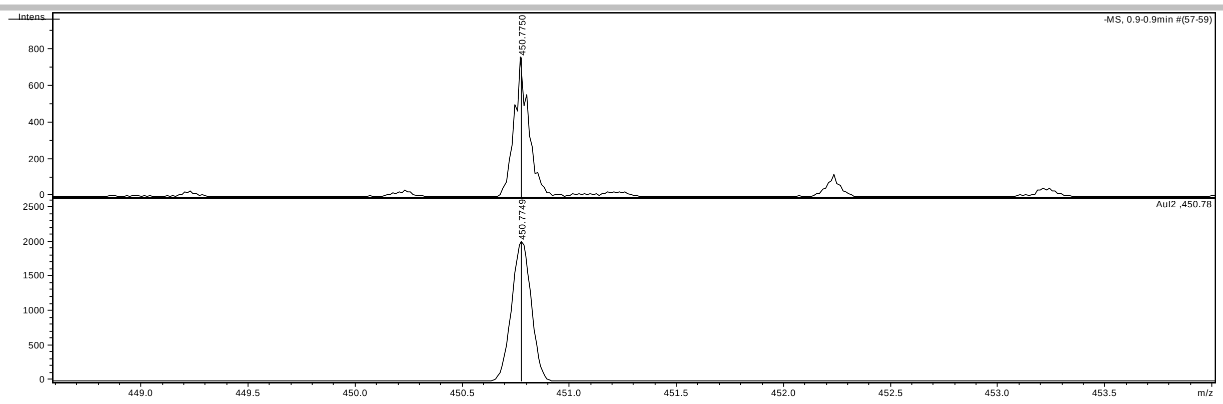

Figure S8: Experimental (*above*) and calculated (*below*) pattern of *m/z* 451 $[AuI_2]^-$ (error 0.081 ppm) corresponding to species **1**.

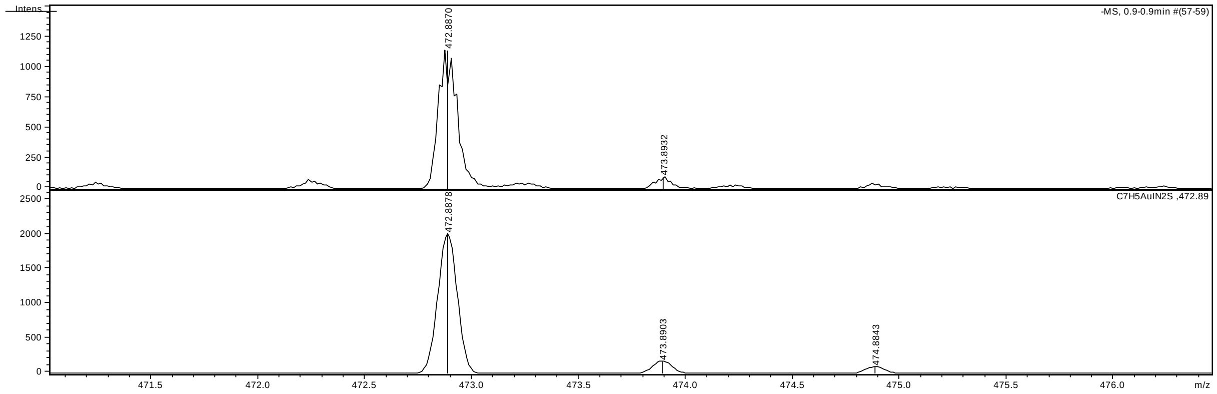

Figure S9: Experimental (*above*) and calculated (*below*) pattern of *m/z* 473 $[(C_7H_6AuIN_2S)\text{-}H]^-$ (error 1.678 ppm) corresponding to species **2**.

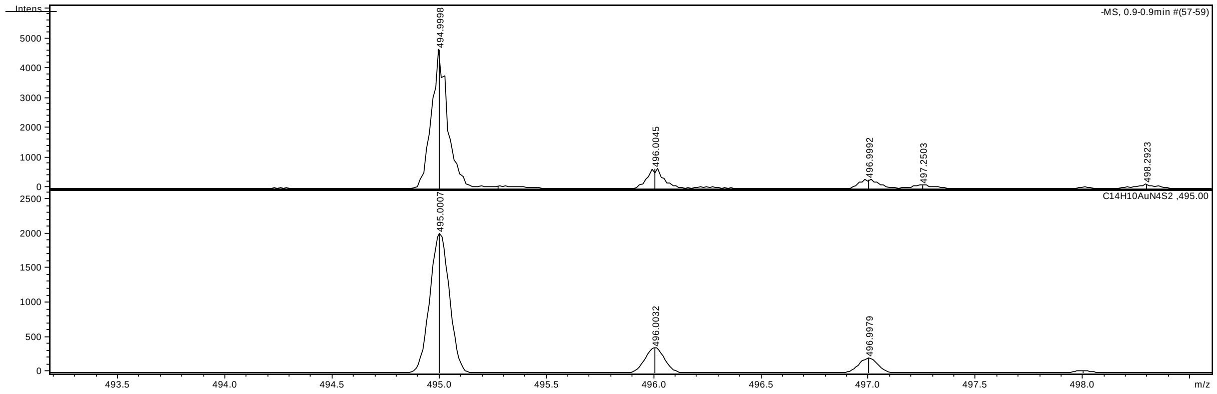

Figure S10: Experimental (*above*) and calculated (*below*) pattern of *m/z* 495 $[(C_{14}H_{12}AuN_4S_2)\text{-}2H]^-$ (error 1.819 ppm) corresponding to species **3**.

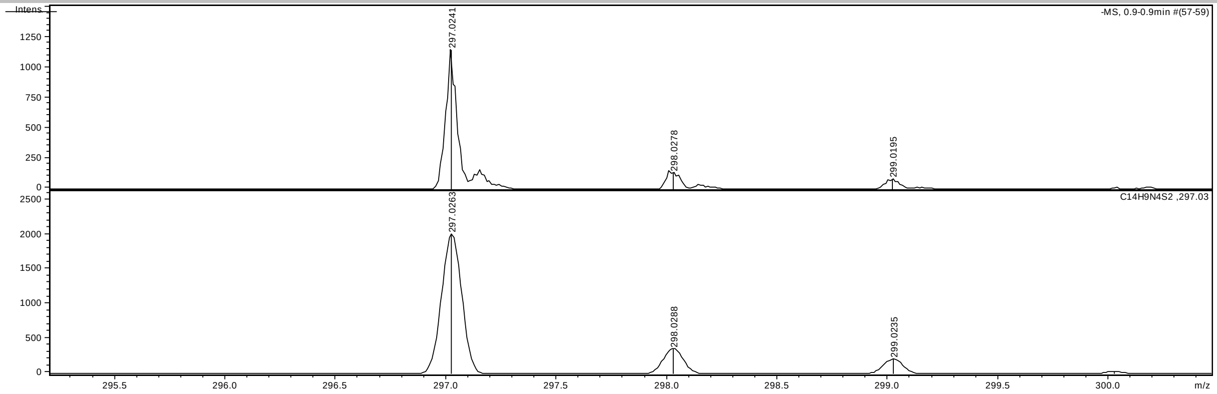

Figure S11: Experimental (*above*) and calculated (*below*) pattern of *m/z* 297 [$(C_{14}H_{10}N_4S_2)$-H]$^-$ (error 7.338 ppm) corresponding to species **5**.

5.2.2. Positive ion mode

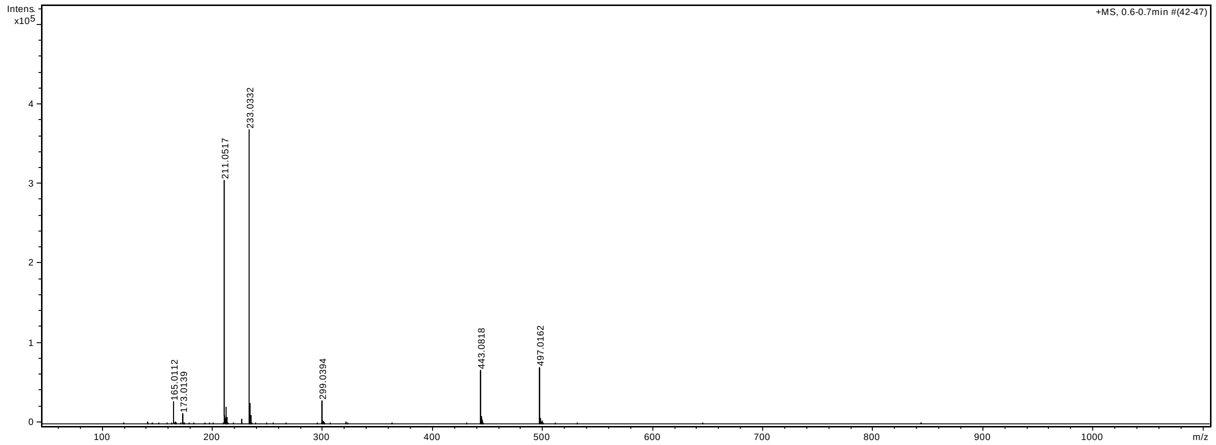

Figure S12: Full range ESI-HRMS spectrum of reaction mixture in positive ion mode (40-1100 m/z). Samples were taken during the standard reaction outlined in section 2.1. Au containing species found: *m/z* 497 $[C_{14}H_{12}AuN_4S_2]^+$, *m/z* 613 $[C_{21}H_{16}AuN_6S_2]^+$.

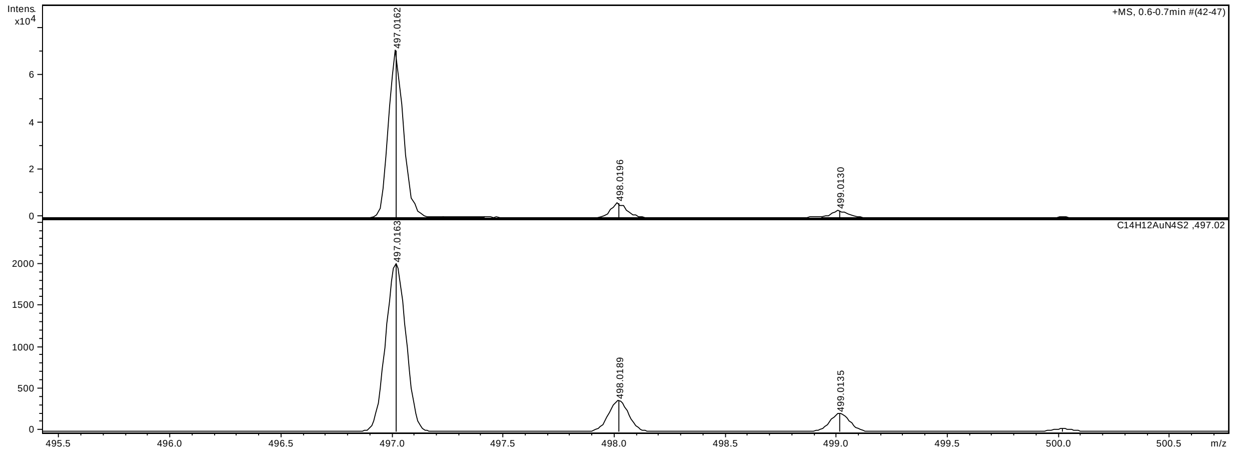

Figure S13: Experimental (*above*) and calculated (*below*) pattern of *m/z* 497 $[C_{14}H_{12}AuN_4S_2]^+$ (error 0.371 ppm) corresponding to species **3**.

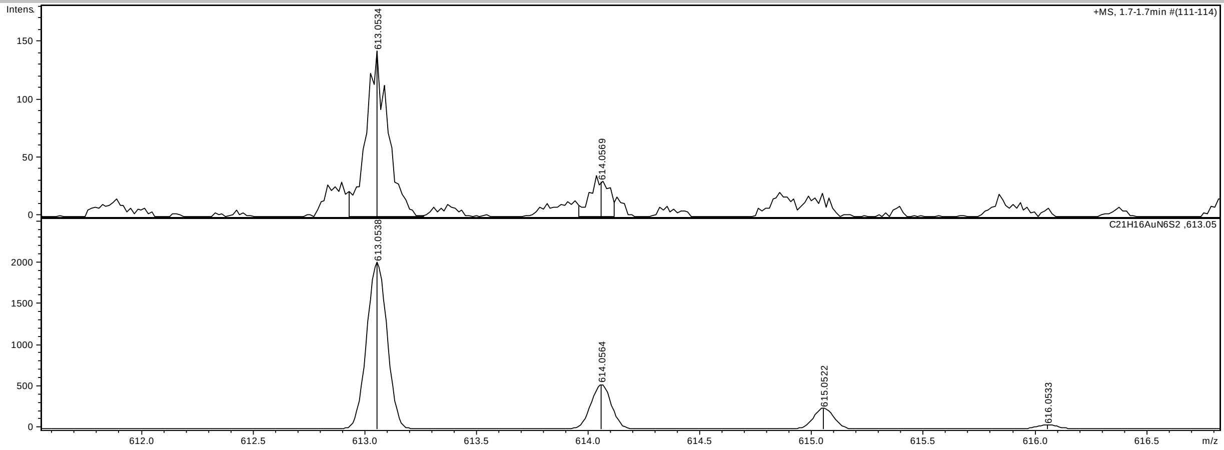

Figure S14: Experimental (*above*) and calculated (*below*) pattern of *m/z* 613 $[C_{21}H_{16}AuN_6S_2]^+$ (error 0.662 ppm) corresponding to species **4**.

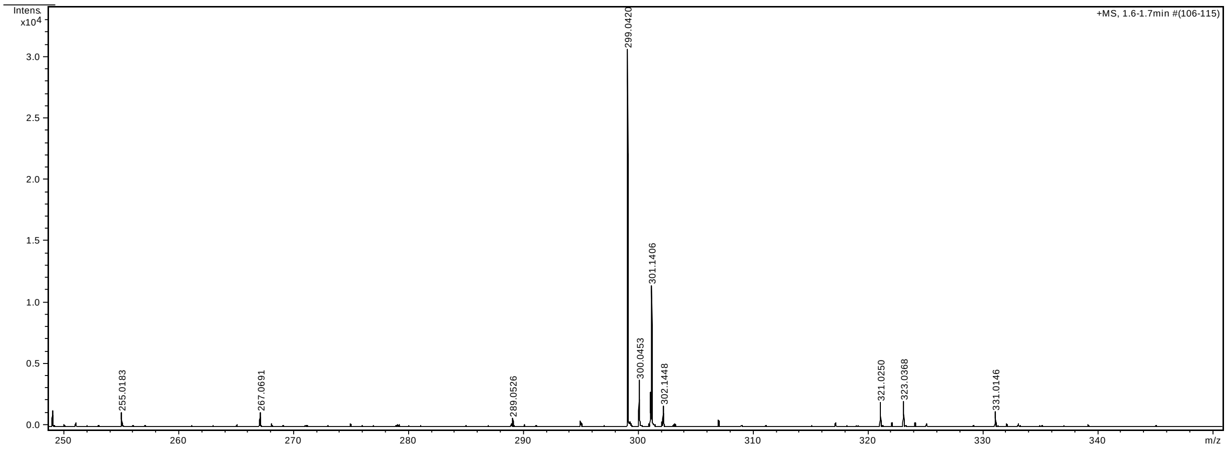

Figure S15: ESI-HRMS spectrum of reaction mixture in positive ion mode (248-350 *m/z*). Samples were taken during the standard reaction outlined in section 2.1. Organic species found: *m/z* 299 $[(C_{14}H_{10}N_6S_2)+H]^+$, *m/z* 267 $[(C_{14}H_{10}N_4S)+H]^+$, *m/z* 331 $[(C_{14}H_{10}N_4S_3)+H]^+$.

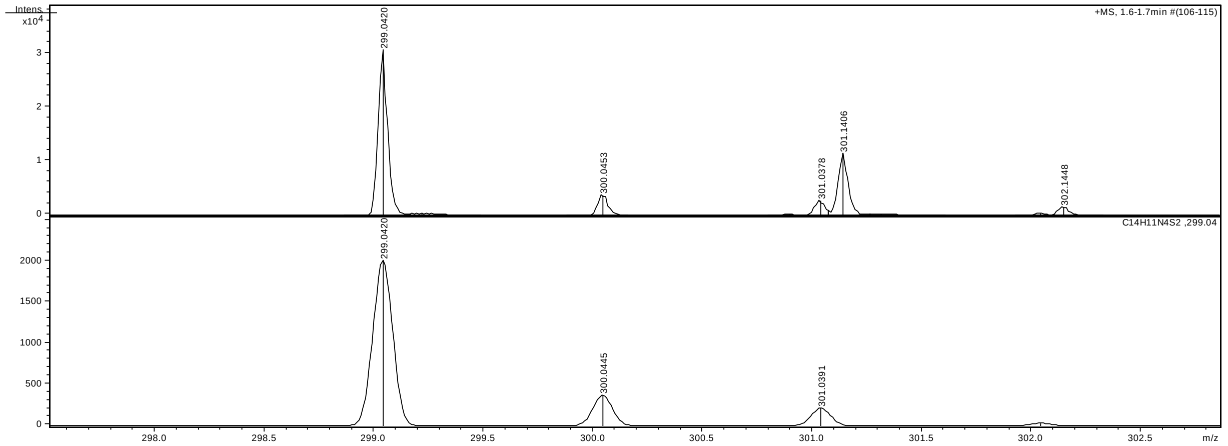

Figure S16: Experimental (*above*) and calculated (*below*) pattern of *m/z* 299 $[(C_{14}H_{10}N_6S_2)+H]^+$ (error 0.001 ppm) corresponding to species **5**.

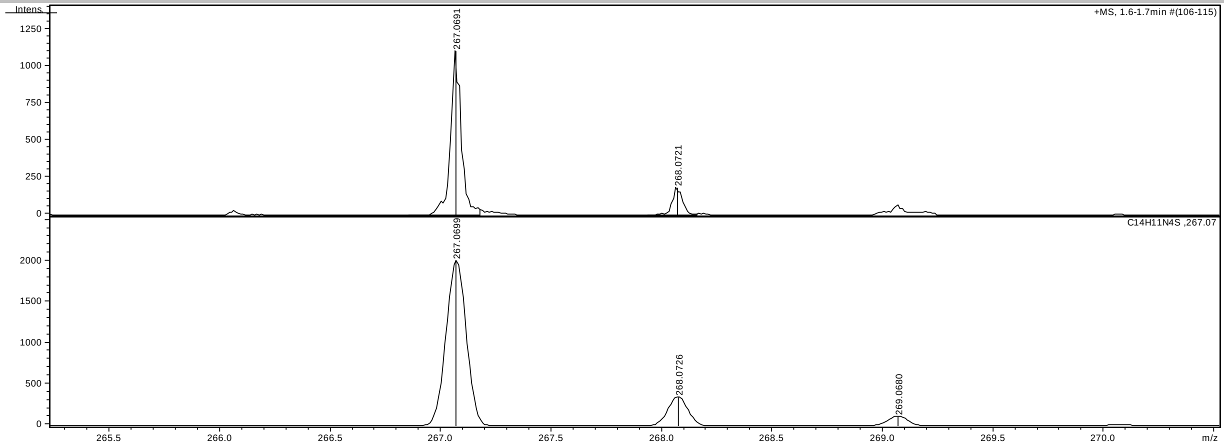

Figure S17: Experimental (*above*) and calculated (*below*) pattern of *m/z* 267 [($C_{14}H_{10}N_4S$)+H]$^+$ (error 2.901 ppm) corresponding to species **6**.

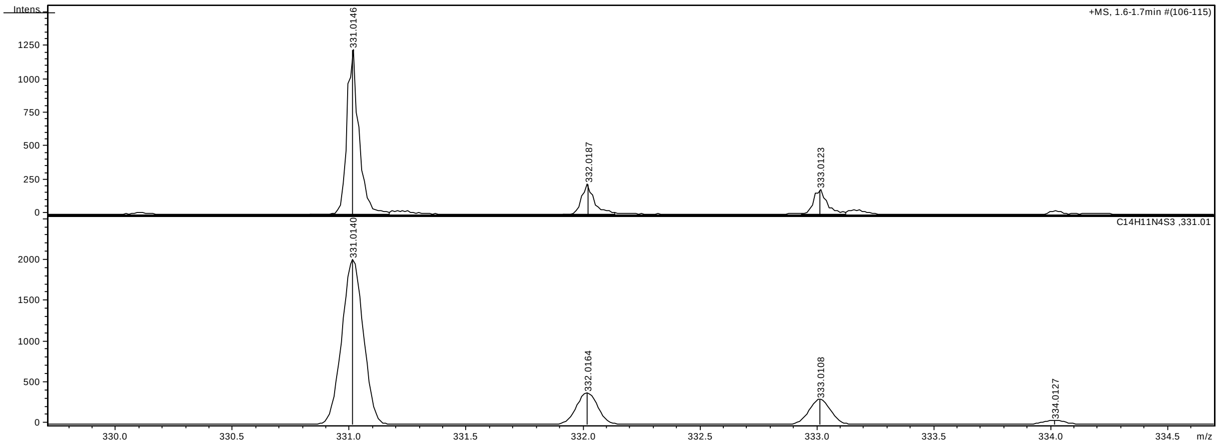

Figure S18: Experimental (*above*) and calculated (*below*) pattern of *m/z* 331 [($C_{14}H_{10}N_4S_3$)+H]$^+$ (error 1.770 ppm) corresponding to species **7**.

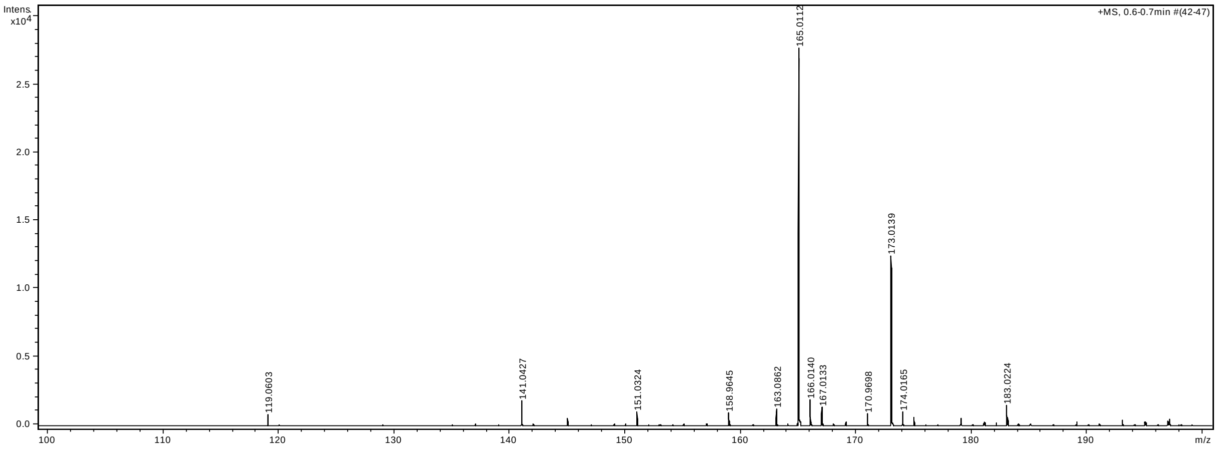

Figure S19: ESI-HRMS spectrum of reaction mixture in positive ion mode (100-200 *m/z*). Samples were taken during the standard reaction outlined in section 2.1. Organic species found: *m/z* 119 [($C_7H_6N_2$)+H]$^+$, *m/z* 151 [($C_7H_6N_2S$)+H]$^+$, *m/z* 173 [($C_7H_6N_2S$)+Na]$^+$.

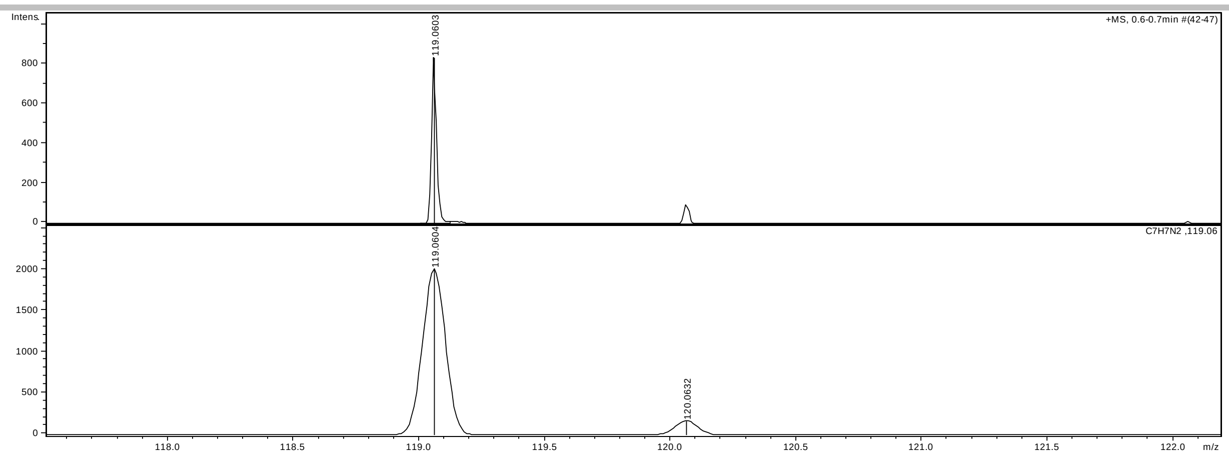

Figure S20: Experimental (*above*) and calculated (*below*) pattern of *m/z* 119 [($C_7H_6N_2$)+H]$^+$ (error 0.446 ppm) corresponding to species **8**.

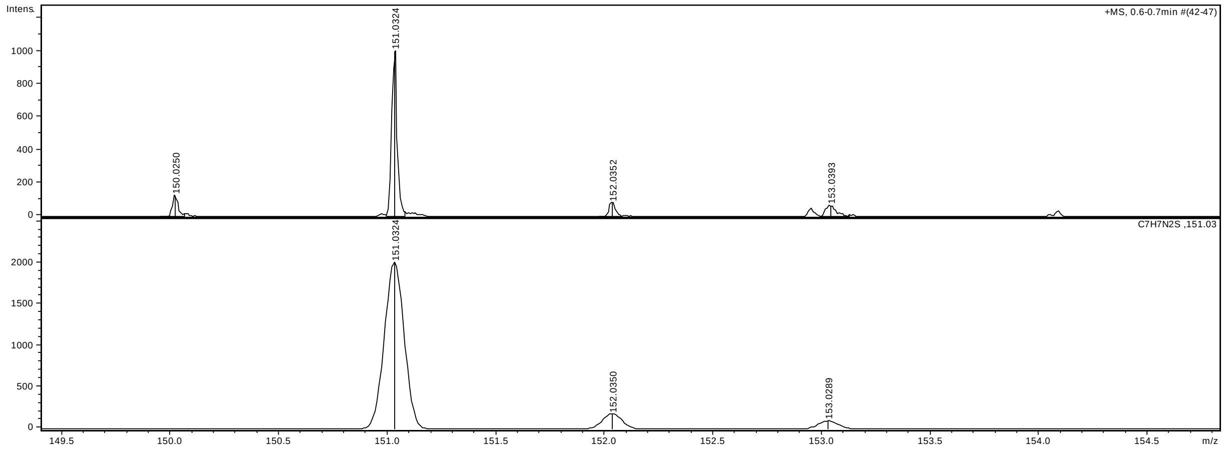

Figure S21: Experimental (*above*) and calculated (*below*) pattern of *m/z* 151 [($C_7H_6N_2S$)+H]$^+$ (error 0.517 ppm) corresponding to 2-MBI.

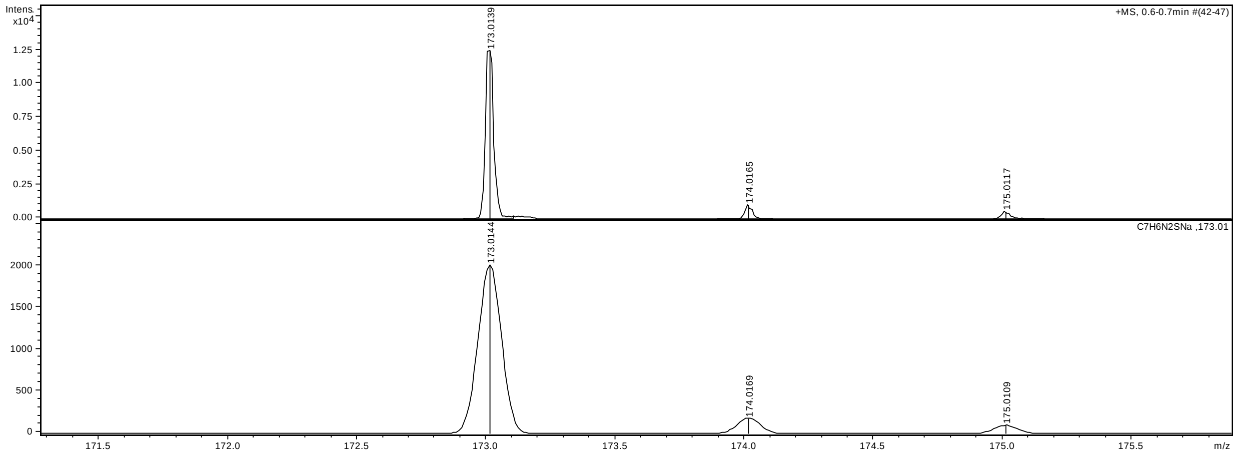

Figure S22: Experimental (*above*) and calculated (*below*) pattern of *m/z* 173 [($C_7H_6N_2S$)+Na]$^+$ (error 2.739 ppm) corresponding to 2-MBI.

5.3. Intensities for selected species vs. time

Graphs of intensities vs. time were plotted for selected species from acquired ESI-HRMS data. All samples were taken from the same experiment. Species **1**, **2** and **3** with corresponding *m/z* values of 451, 473 and 495, respectively, were followed for the first three hours of the reaction (Figure S23). Samples were prepared three times more concentrated than outlined in Section 5.1.

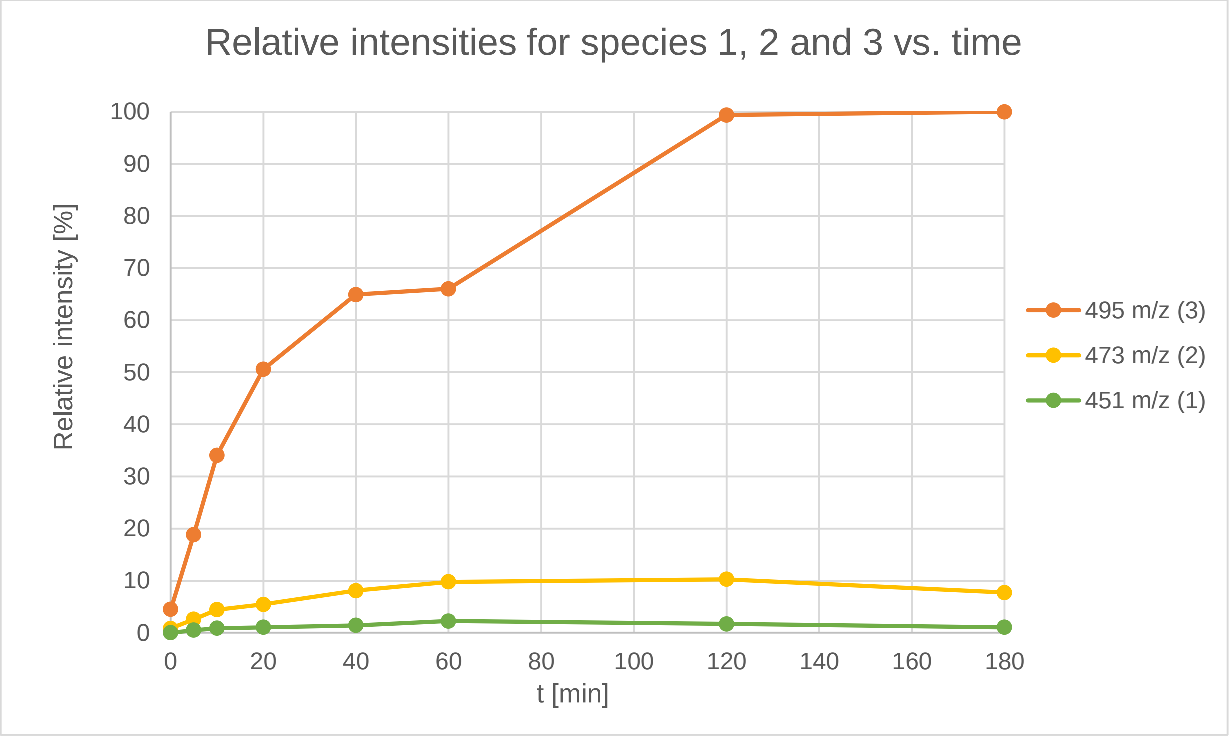

Figure S23: Relative intensities for *m/z* 451, 473 and 495 (negative ion mode) vs. time. The highest intensity was set to 100% and other data points were adjusted accordingly.

6. Recycling of Au and 2-MBI

6.1. Procedure

After quantitative dissolution of Au with the scale-up procedure (20 mg of Au, see Section 2.2), reaction mixture was placed in an ice bath to cool for 15 min. Then, $NaBH_4$ (151.3 mg, 4 mmol) was slowly added during a 40 min period. Reaction mixture was left at 0°C for an additional 5 min before stirring vigorously at room temperature for 4 h. Formation of black precipitate was noted. Next, water (30 mL) was added, and the reaction mixture was left to stir. Previously precipitated black particles coagulated to form black flakes, which were then collected by filtration by using Büchner funnel. Precipitate was washed in the following order: with water, H_2SO_4 (aq), water, NaOH (aq), water, distilled water, EtOH and finally with Et_2O. The filtrate was kept for 2-MBI ligand recycling. The precipitate was dried under reduced pressure (vacuum pump) to afford 18.4 mg of black powder, later proven to be elemental Au by FESEM-EDS analysis (yield=92%). Flask remained loosely closed with plastic stopper throughout the whole reduction process.
Solvents from EtOH/water filtrate were removed under reduced pressure and 10% aqueous HCl was added to quench the residual $NaBH_4$. Formed precipitate was collected by filtration and washed with water, distilled water and Et_2O, respectively, to afford 123.9 mg (yield=41%) of white crystalline material characterized by NMR to be pure 2-MBI[1] (Figures S24 and S25).

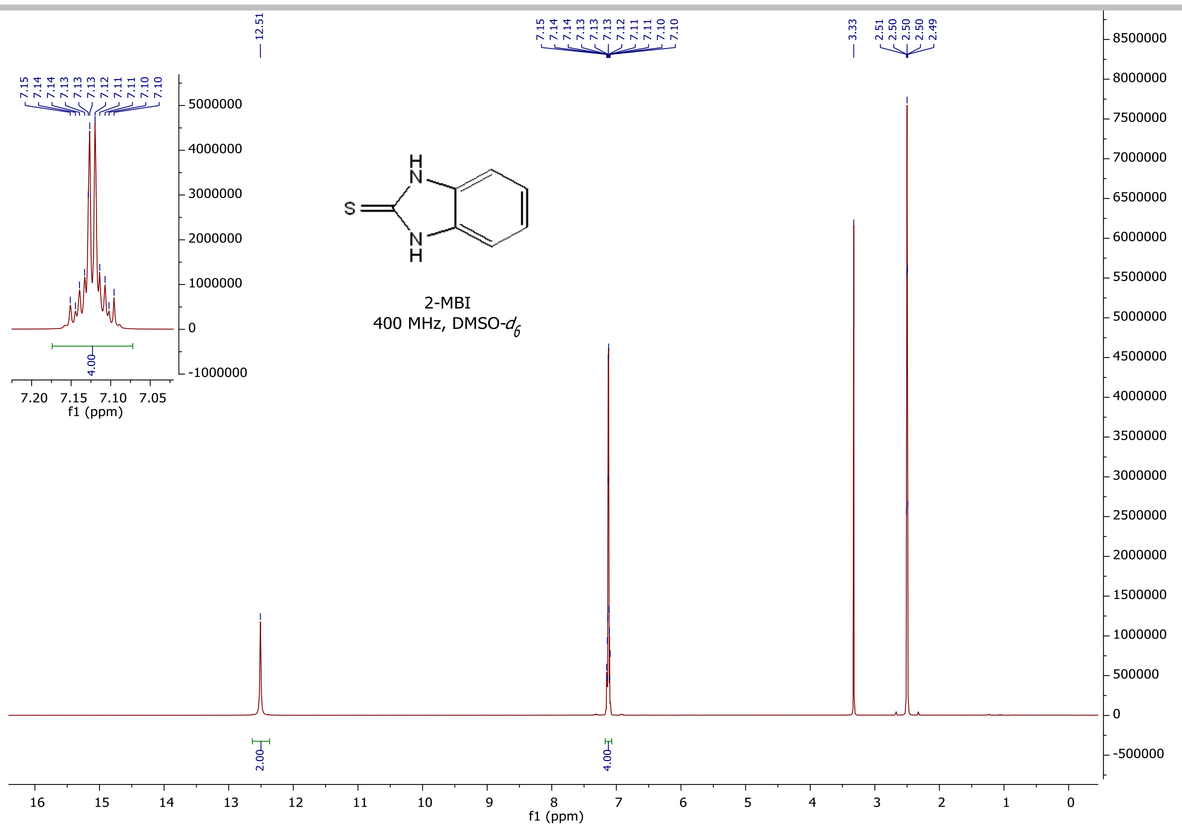

Figure S24: ^{1}H NMR spectrum of isolated 2-MBI from scale-up Au dissolution reaction after reduction (400 MHz, DMSO-d_6). Chemical shifts δ: 12.51 (s, 2H, N*H*), 7.18 – 7.08 (m, 4H).

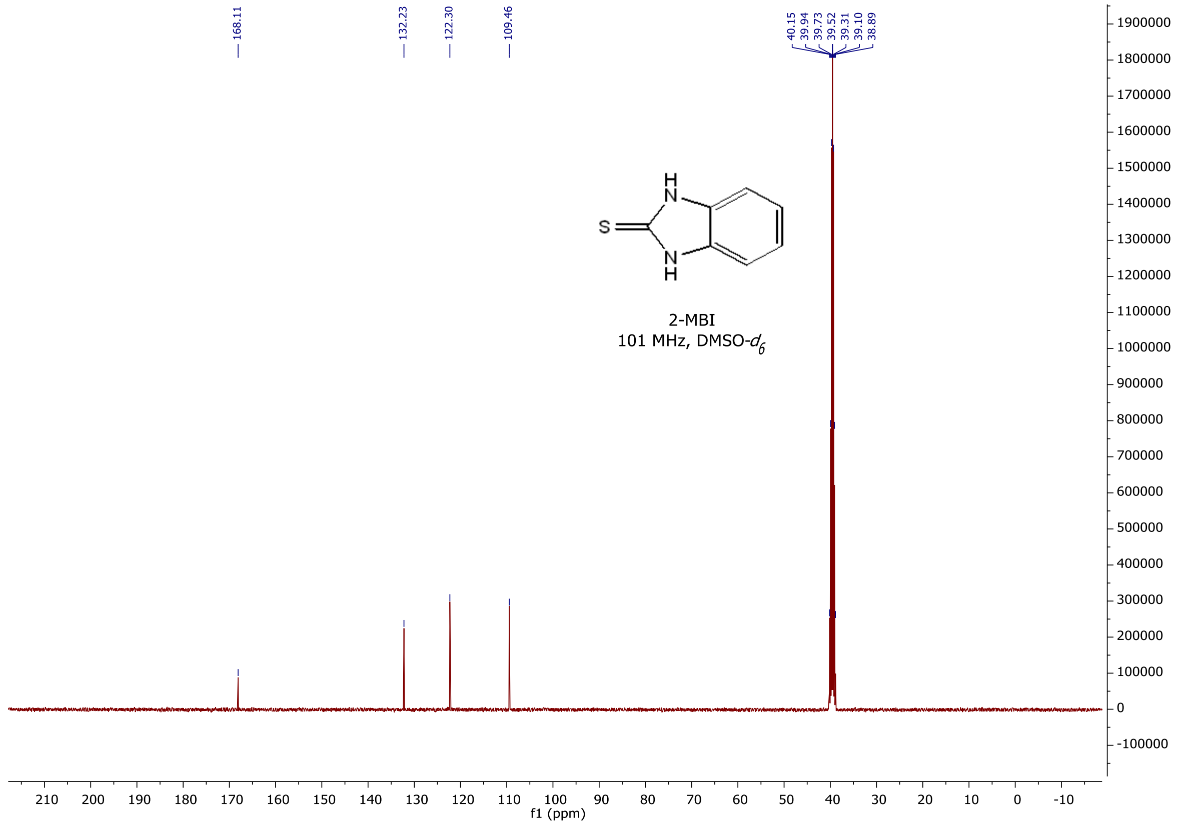

Figure S25: ^{13}C NMR spectrum of isolated 2-MBI from scale-up Au dissolution reaction after reduction (101 MHz, DMSO-d_6). Chemical shifts δ: 109.5, 122.3, 132.2, 168.1.

6.2. FESEM-EDS analysis

An Oxford INCA 350 energy-dispersive X-ray microanalysis system connected with a Hitachi S-4800 field emission scanning electron microscope (FESEM) was used for the energy-dispersive X-ray spectrometry (EDS) measurements. Au sample was washed and dried before analysis as described above. As seen from Figure S26, precipitate acquired was pure Au with particle size 10-20 nm in diameter.

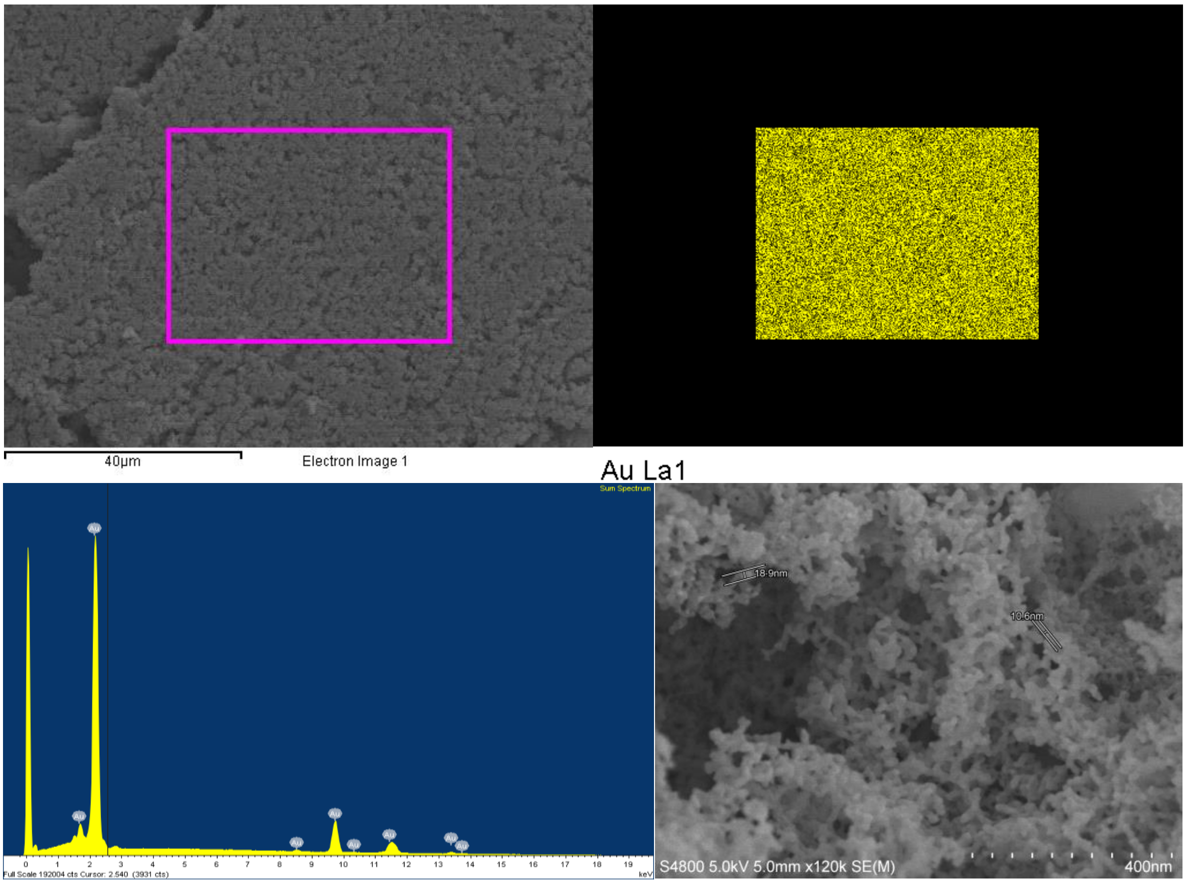

Figure S26: FESEM-EDS analysis of precipitated Au powder. SEM image of Au powder (*above, left*). Au mapping for pink square area (*above, right*). Au mapping spectrum (*below, left*). Zoomed SEM image of Au particle (*below, right*).

6.3. NMR study of reaction mixture before and after reduction

^{1}H NMR experiments were conducted to investigate existing species before and after reduction. Sample was taken from scale-up reaction after dissolution. Solvent from sample was removed under reduced pressure and residue was dissolved in DMSO-d_6 for ^{1}H NMR analysis (Figure S27). After reduction of scale-up reaction with $NaBH_4$, solvent was evaporated under reduced pressure and residue was dissolved in EtOAc. Solution was transported to separating funnel and saturated NaCl (aq) was added. Mixture was extracted 4 times with EtOAc, organic fractions combined and dried over anhydrous $MgSO_4$ before EtOAc was removed under reduced pressure. Sample was again dissolved in DMSO-d_6 for ^{1}H NMR analysis (Figure S28).
As seen from Figure S27, after Au dissolution the ^{1}H NMR peaks can be assigned to 2-MBI[1], thioether **6** (δ 7.18 – 7.20, 7.58 – 7.60)[2] and disulphide **5** (broad peaks at δ 7.33 and 7.76)[3]. Integral values suggest that another species similar to **5** and **6** is present in the reaction mixture – broad peaks at δ 7.33 and 7.59 could be attributed to trisulfide **7**. Mentioned peaks disappear after the reduction, which can be another proof of **7** as well as conformation from ESI-HRMS.

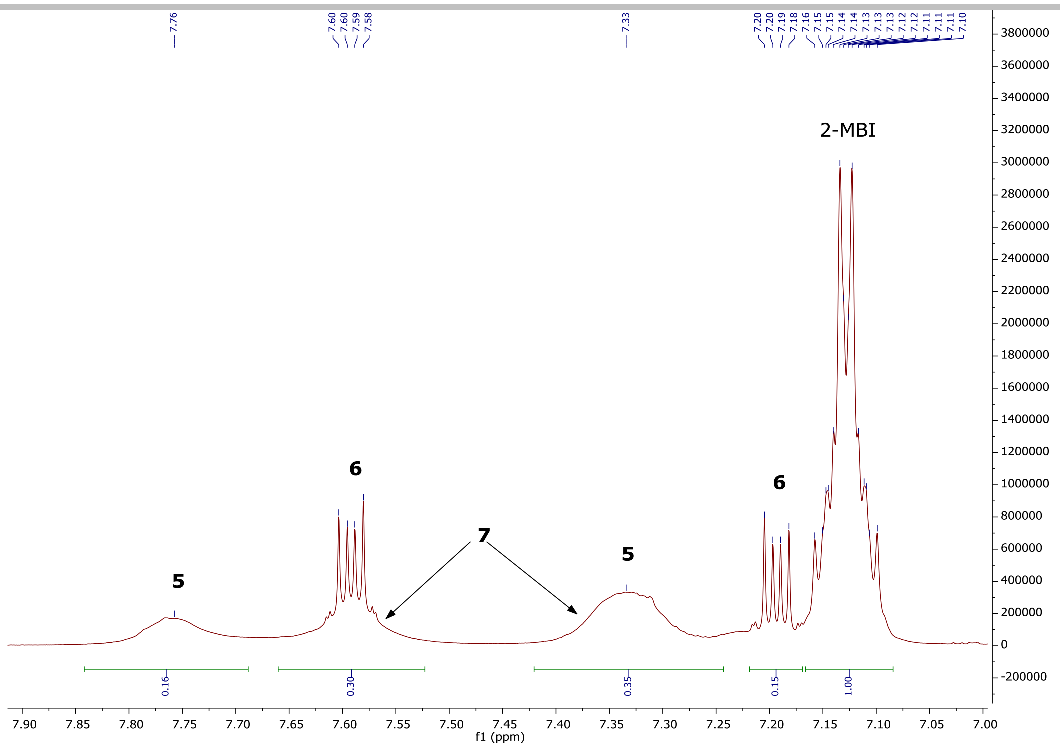

Figure S27: Aromatic part of ^{1}H NMR spectrum (400 MHz, DMSO-d_6) after dissolution of Au (before reduction). Peaks are assigned to species **5**, **6**, **7** and 2-MBI.

Figure S28 shows that after reduction, only thioether **6** and 2-MBI remain in the reaction mixture.

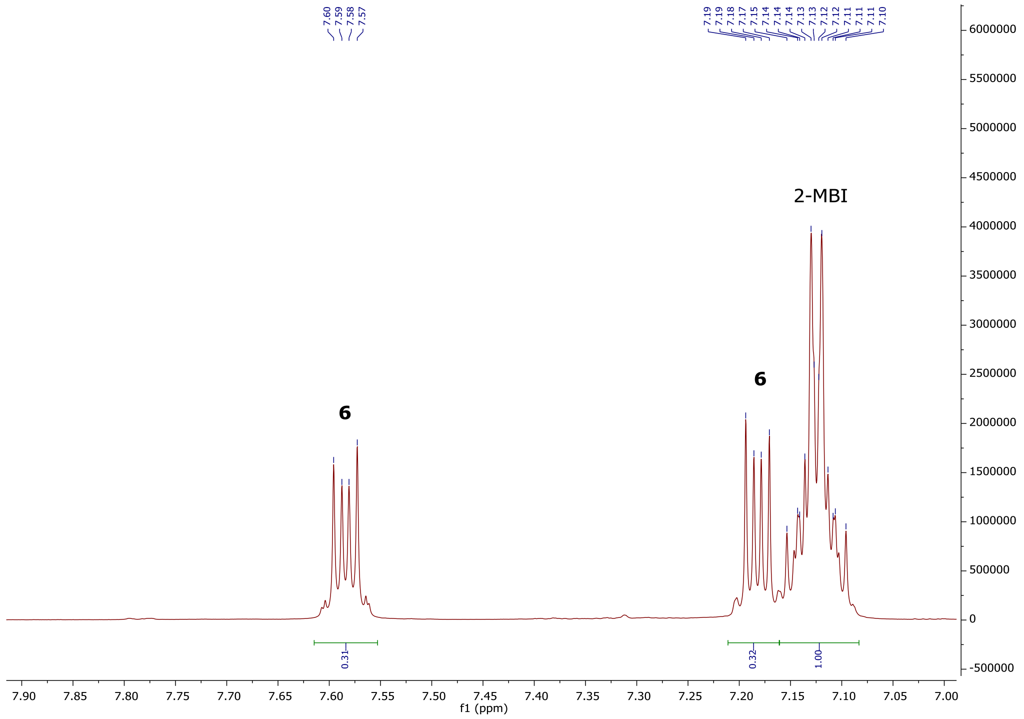

Figure S28: Aromatic part of ^{1}H NMR spectrum (400 MHz, DMSO-d_6) of scale-up reaction mixture after reduction. Peaks are assigned to species **6** and 2-MBI.

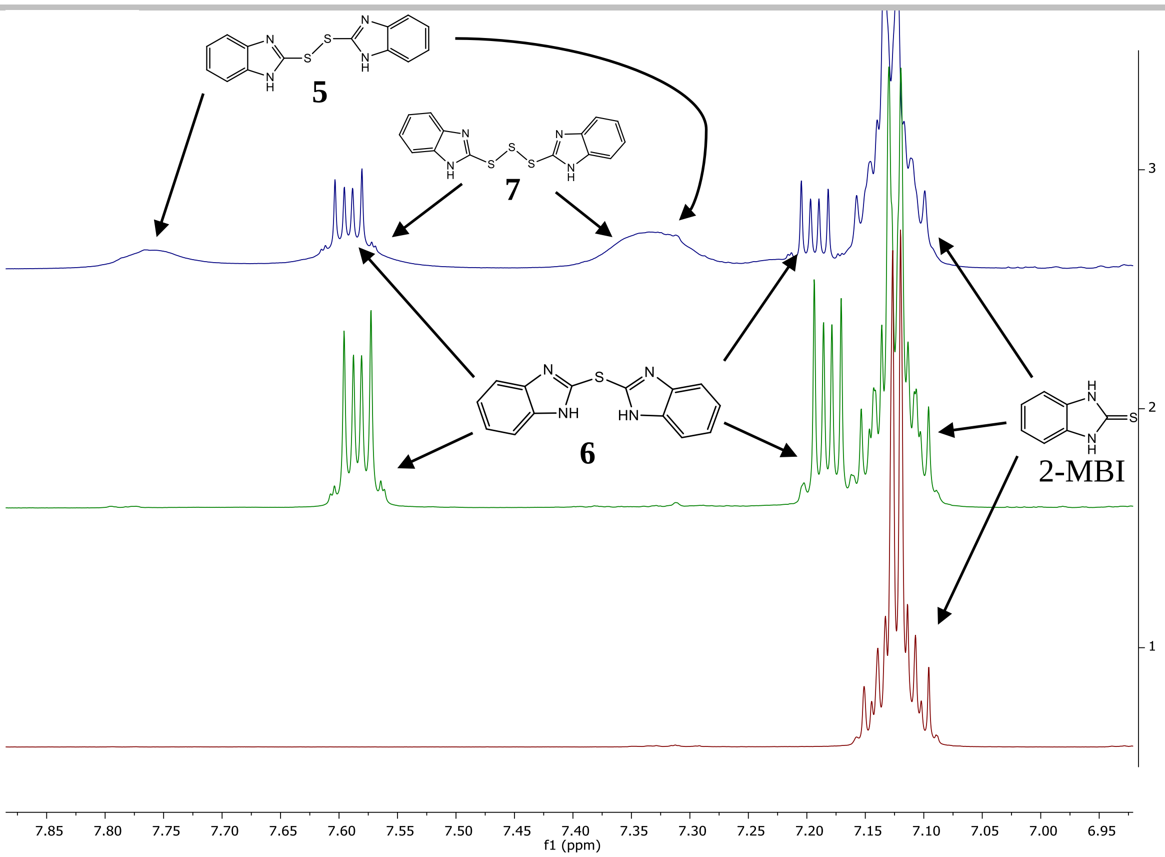

Figure S29: Comparison between aromatic regions of ^{1}H NMR spectra of reaction mixture before reduction (*above*), after reduction (*middle*) and isolated 2-MBI (*below*).

As seen from Figure S29, peaks assigned to disulphide **5** and trisulphide **7** disappear after reduction. **5** and **7** are reduced to 2-MBI, whereas partial transformation to thioether **6** takes place as noted from comparing integrals between spectra depicted in Figure S27 and Figure S28.

7. Computational details

All calculations were performed using ORCA 5.0.[4] Structures were optimized using the TPSS[5] functional with def2-TZVP basis set[6] and DFT with standard integration grids. Weak interactions were accounted for using the D3 dispersion correction with Becke-Johnson damping[7]. Solvation effects were accounted using the conduction conductor-like polarizable continuum model, the CPCM solvation model[8] with 24.3 (ethanol) dielectric constant. Thermal corrections at 60°C were obtained by calculating harmonic vibrational frequencies for all structures at the TPSS-D3/def2-TZVP level, and chemical potentials (c.p.) were obtained using the quasi-rigid rotor harmonic oscillator (quasi-RRHO) approach proposed by Grimme.[9] The quasi-RRHO approach uses the free-rotor entropy for all modes with frequencies below 35 cm-1, while the standard RRHO approach is used for other modes. In addition, the harmonic vibrational frequencies have been scaled by a factor of 0.9914. The Gibbs free energies are then obtained as summation of the zero point energy and chemical potentials.

7.1 Calculation of ΔG for substitution reactions

The change of Gibbs free energy (ΔG) for substitution reactions from **1** to **2** and from **2** to **3** were determined as difference between Gibbs energies of products and reactants (Figure S30). When I^- and I_2 are both present in the solution, I_3^- is readily formed with a large stability constant in ethanol. I_3^- was considered when calculating ΔG for substitution reactions when I_2 concentration was high.

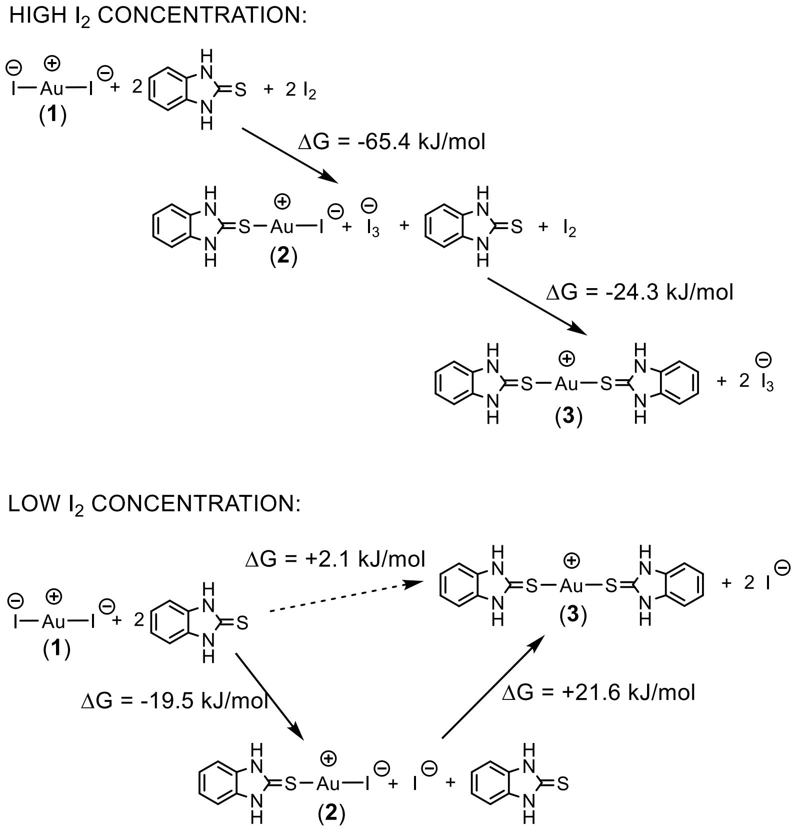

Figure S30: Substitution reactions from **1** to **2** and from **2** to **3** and calculated changes of free Gibbs energies (ΔG) when I_2 concentration is high and low.

7.2 Calculated energies and cartesian coordinates for different species

All structures are optimized at TPSS-D3/def2-TZVP level. Energies are in *Hartrees*.

Species I^-:

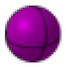

Zero Point Energy (Hartree)	0.0000000000
Inner Energy (Hartree)	-297.7784859152
Enthalpy (Hartree)	-297.7774308649
Electronic entropy	0.0000000000
Rotational entropy	0.0000000000
Vibrational entropy	0.0000000000
Translational entropy	0.0000000000
Entropy	0.0217563100
Gibbs Energy (Hartree)	-297.7991871749

Number of atoms: 1
Coordinates:
0 I -1.821780000000 -0.158420000000 0.000000000000

Species I_2:

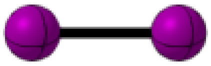

Zero Point Energy (Hartree)	0.0004818158
Inner Energy (Hartree)	-595.2149161499
Enthalpy (Hartree)	-595.2138610997
Electronic entropy	0.0000000000
Rotational entropy	0.0102844709
Vibrational entropy	0.0011862164
Translational entropy	0.0102844709
Entropy	0.0343239224
Gibbs Energy (Hartree)	-595.2481850221

Number of atoms: 2			
Coordinates:			
0 I	-6.356374997596	1.346530000000	0.000000000000
1 I	-3.667985002404	1.346530000000	0.000000000000

Species I_3^-:

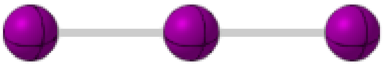

Zero Point Energy (Hartree)	0.0007871190
Inner Energy (Hartree)	-893.0232216085
Enthalpy (Hartree)	-893.0221665583
Electronic entropy	0.0000000000
Rotational entropy	0.0119525609
Vibrational entropy	0.0072463487
Translational entropy	0.0119525609
Entropy	0.0426938047
Gibbs Energy (Hartree)	-893.0648603630

Number of atoms: 3			
Coordinates:			
0 I	-8.813010860572	2.257430004298	-0.000000016106
1 I	-5.849494893798	2.257429992720	0.000000030665
2 I	-2.886164245630	2.257430002982	-0.000000014560

Species 2-MBI:

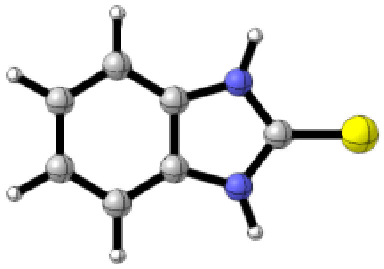

Zero Point Energy (Hartree)	0.1191635556
Inner Energy (Hartree)	-778.2387965003
Enthalpy (Hartree)	-778.2377414500
Electronic entropy	0.0000000000
Rotational entropy	0.0152010305
Vibrational entropy	0.0097863028
Translational entropy	0.0152010305
Entropy	0.0470103970
Gibbs Energy (Hartree)	-778.2847518471

Number of atoms: 16			
Coordinates:			
0 C	-3.227518017926	-1.436939062028	0.000000009414
1 C	-3.230128075402	1.429912546706	0.000000017128
2 C	-4.419109023131	-0.706735466924	-0.000000023554
3 C	-2.042308225939	-0.707085643089	0.000000004712
4 C	-2.043587553606	0.702226569294	0.000000012939
5 C	-4.420384495115	0.697529247006	-0.000000006938
6 N	-0.709300189090	1.088285032216	0.000000012787
7 C	0.122904648061	-0.000474243719	-0.000000035305
8 N	-0.707322919100	-1.090737128564	0.000000020148
9 S	1.803571078867	0.001054203689	-0.000000055201
10 H	-3.223198013003	-2.521965536360	0.000000011878
11 H	-3.227798769430	2.514944825014	0.000000029307
12 H	-5.365184694885	-1.239414091856	-0.000000023521
13 H	-5.367430225539	1.228481282243	-0.000000027280
14 H	-0.362098163327	2.039308879669	0.000000011724
15 H	-0.358407361436	-2.041131413297	0.000000041762

Species **1**, $[Au^{I}I2]^{-}$:

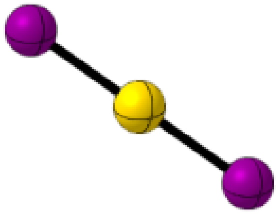

Zero Point Energy (Hartree)	0.0008945102
Inner Energy (Hartree)	-731.1921429297
Enthalpy (Hartree)	-731.1910878794
Electronic entropy	0.0000000000
Rotational entropy	0.0041721037
Vibrational entropy	0.0047632272
Translational entropy	0.0041721037
Entropy	0.0326975749
Gibbs Energy (Hartree)	-731.2237854543

Number of atoms: 3
Coordinates:
0 Au 0.633337000000 0.895688000000 -0.000000000000
1 I -1.477294000000 2.388095000000 0.000000000000
2 I 2.743957000000 -0.596782000000 0.000000000000

Species **2**, [Au^{I}I(2-MBI)]:

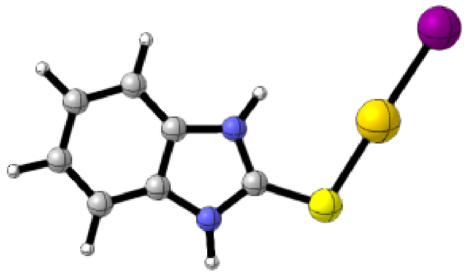

Zero Point Energy (Hartree)	0.1212219202
Inner Energy (Hartree)	-1211.6555875167
Enthalpy (Hartree)	-1211.6545324664
Electronic entropy	0.0000000000
Rotational entropy	0.0183550389
Vibrational entropy	0.0200570744
Translational entropy	0.0183550389
Entropy	0.0622541105
Gibbs Energy (Hartree)	-1211.7167865769

Number of atoms: 18			
Coordinates:			
0 C	0.769587000000	2.763564000000	0.000001000000
1 C	-0.003507000000	-0.002503000000	0.000001000000
2 C	-0.571300000000	2.377477000000	-0.000009000000
3 C	1.712131000000	1.739275000000	0.000011000000
4 C	1.333925000000	0.383877000000	0.000011000000
5 C	-0.949447000000	1.023277000000	-0.000010000000
6 N	2.517927000000	-0.348660000000	0.000023000000
7 C	3.588957000000	0.485677000000	0.000026000000
8 N	3.104128000000	1.751223000000	0.000019000000
9 S	5.220906000000	-0.032079000000	0.000029000000
10 Au	6.448486000000	1.927671000000	-0.000135000000
11 I	7.861240000000	4.083318000000	0.000025000000
12 H	1.066768000000	3.806713000000	0.000000000000
13 H	-0.292109000000	-1.048113000000	-0.000000000000
14 H	-1.341209000000	3.142698000000	-0.000018000000
15 H	-2.004524000000	0.767958000000	-0.000018000000
16 H	2.591786000000	-1.359610000000	0.000026000000
17 H	3.704376000000	2.570186000000	0.000019000000

Species **3**, $[Au^{I}(2\text{-}MBI)_2]^{+}$:

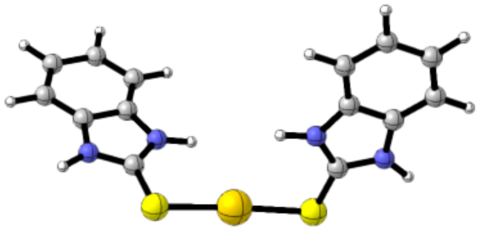

Zero Point Energy (Hartree)	0.2415730718
Inner Energy (Hartree)	-1692.1152621074
Enthalpy (Hartree)	-1692.1142070572
Electronic entropy	0.0000000000
Rotational entropy	0.0194049920
Vibrational entropy	0.0365809158
Translational entropy	0.0194049920
Entropy	0.0799038310
Gibbs Energy (Hartree)	-1692.1941108882

Number of atoms: 33
Coordinates:
0 C -0.803559685902 -0.136907128137 2.105996703305
1 C 1.418001592724 -1.751727607640 2.949843713846
2 C -0.124609622607 0.115090663858 3.298250463024
3 C -0.348121352863 -1.211387413367 1.346661269648
4 C 0.739454098413 -2.002146292277 1.760259832422
5 C 0.963089858272 -0.675179111287 3.711219310654
6 N 0.915272313915 -2.966096601818 0.771449931601
7 C -0.001823500396 -2.802875141776 -0.214187355788
8 N -0.769639766319 -1.741919175818 0.129947377053
9 S -0.103005655758 -3.800207282699 -1.606350574220
10 Au -1.803667402921 -2.895140583797 -2.874200270291
11 N -1.588310953524 -0.354411439308 -5.072268935032
12 C -1.452207608739 0.701327795789 -5.969678179687
13 C -2.713316649808 0.865538417583 -6.571971495475
14 N -3.541284067259 -0.099239616057 -6.004496949126
15 C -2.853841405769 -0.834961582994 -5.096216560777
16 C -2.944006428766 1.845848134897 -7.533182059566
17 C -1.859566620110 2.657069949441 -7.867568687239
18 C -0.599287556936 2.492924403411 -7.265300966616
19 C -0.369201136744 1.509937100532 -6.302526122388
20 S -3.536075039750 -2.116663330461 -4.183295649054
21 H -1.642499217862 0.470182764459 1.783560730652
22 H 2.256519850695 -2.363561646283 3.264441827095
23 H -0.444727170994 0.943044003750 3.922972305840
24 H 1.461934801455 -0.441820579982 4.646619059030
25 H 1.623434869377 -3.692588034179 0.768222219494
26 H -1.540365549381 -1.406008951542 -0.438512688514
27 H -0.864123554692 -0.732583329301 -4.469980404450
28 H -4.520135222996 -0.248352908227 -6.223940350063
29 H -3.917228239273 1.969561215051 -7.995704526743
30 H -1.993052886151 3.435175702070 -8.612554164128
31 H 0.216004279649 3.147522749780 -7.556897782860
32 H 0.601025631019 1.379556856327 -5.835609021652

References

[1] W. Li, H. Zheng, *Org. Prep. Proced. Int.* **2019**, *51:2*, 175–181.
[2] P. Manivel, K. Prabakaran, V. Krishnakumar, F.-R. Nawaz Khan, T. Maiyalagan, *Ind. Eng. Chem. Res.* **2014**, *53*, 7866−7870.
[3] F. Shirini, M. A. Zolfigol, M. Khaleghi, *Mendeleev Commun.* **2004**, *14(1)*, 34–35.
[4] F. Neese, F. Wennmohs, U. Becker, C. Riplinger, *J. Chem. Phys.* **2020**, *152*, 224108.
[5] J. Tao, J. P. Perdew, V. N. Staroverov, G. E. Scuseria, *Phys. Rev. Lett.* **2003**, *91*, 146401.
[6] F. Weigend, R. Ahlrichs, *Phys. Chem. Chem. Phys.* **2005**, *7*, 3297–3305.
[7] S. Grimme, S. Ehrlich, L. Goerigk, *J. Comput. Chem.* **2011**, *32*, 1456–1465.
[8] V. Barone, M. Cossi, *J. Phys. Chem. A* **1998**, *102*, 1995–2001.
[9] S. Grimme, *Chem. Eur. J.* **2012**, *18*, 9955–9964.

Author Contributions

Anže Zupanc: investigation, methodology, data curation, formal analysis, software, writing – original draft, and writing – review and editing; Eeva Heliövaara: data curation, formal analysis, supervision, and writing – review and editing; Karina Moslova: resources, and ESI-HRMS analyses; Aleksi Eronen: data curation, formal analysis, and software; Marianna Kemell: resources, SEM and EDS analyses; Črtomir Podlipnik: DFT calculations; Prof. Marjan Jereb: methodology and data curation; Prof. Timo Repo*: supervision and writing – review and editing.
